# Hsa-microRNA-1249-3p/Homeobox A13 axis modulates the expression of β-catenin gene in human epithelial cells

**DOI:** 10.1038/s41598-023-49837-0

**Published:** 2023-12-18

**Authors:** Chiara Mazziotta, Maria Rosa Iaquinta, Maria Letizia Tramarin, Giada Badiale, Christian Felice Cervellera, Giulia Tonnini, Simone Patergnani, Paolo Pinton, Giovanni Lanza, Roberta Gafà, Mauro Tognon, Fernanda Martini, Monica De Mattei, John Charles Rotondo

**Affiliations:** 1 Department of Medical Sciences, University of Ferrara, 64/b, Fossato di Mortara Street, 44121 Ferrara, Italy; 2 Center for Studies on Gender Medicine, Department of Medical Sciences, University of Ferrara, 44121 Ferrara, Italy; 3 Laboratory for Technologies of Advanced Therapies (LTTA), University of Ferrara, 44121 Ferrara, Italy; 4 Department of Translational Medicine, University of Ferrara, 44121 Ferrara, Italy

**Keywords:** Cancer, Cell biology, Molecular biology

## Abstract

Intercellular adhesion is a key function for epithelial cells. The fundamental mechanisms relying on epithelial cell adhesion have been partially uncovered. Hsa-microRNA-1249-3p (hsa-miR-1249-3p) plays a role in the epithelial mesenchymal transition in carcinoma cells, but its physiological function in epithelial cells is unknown. We aimed to investigate the role and molecular mechanisms of hsa-miR-1249-3p on epithelial cell functions. Hsa-miR-1249-3p was overexpressed in human epithelial cells and uterine cervical tissues, compared to cervical carcinoma cells and precancerous tissues, respectively. Hsa-miR-1249-3p was analyzed to verify its regulatory function on Homeobox A13 (HOXA13) target gene and its downstream cell adhesion gene β-catenin. Functional experiments indicated that hsa-miR-1249-3p inhibition prompted the mRNA and protein overexpression of HOXA13 which, in turn, led to the β-catenin protein expression. Moreover, hsa-miR-1249-3p inhibition induced a strong colony forming ability in epithelial cells, suggesting the miR involvement in cell adhesion machinery. These data indicate that hsa-miR-1249-3p regulates the expression of HOXA13 and its downstream cell adhesion gene β-catenin, possible resulting in cell adhesion modification in epithelial cells. This study will allow the set-up of further investigations aimed at exploring the relationship between the hsa-miR-1249-3p/HOXA13 axis and downstream cell adhesion genes.

## Introduction

Cell adhesion is a dynamic and stable process that supports tissue morphogenesis and spatial organization^1,2^. Different cell behaviors and functions, such as proliferation, development, survival, differentiation, migration and tissue formation are related to cell adhesion^3^. Therefore, the loss of integrity of cell adhesion contacts may contribute to the occurrence of diseases, including cancer.

The maintenance of proper intercellular interactions and functions of epithelial cells is mainly mediated by different families of adhesion molecules including integrins, cadherins, selectins, as well as α-catenin, β-catenin and vinculin^4^. Growing evidence indicates that microRNAs (miRNAs) can regulate the expression of genes implicated in the adhesion machinery of epithelial cells^5^.

miRNAs are short (18–25 nucleotide [nt]) single strand non-coding RNA molecules, which have a key role in the post-transcriptional regulation of multiple genes^6^. miRNAs exert their activity by binding to the 3 ′untranslated region (3′UTR) of a target protein-coding mRNA, leading to the negative regulation of gene expression^7^.

miRNAs are important determinants for epithelial cell functions. They play a key role during the embryonic epithelial tissue morphogenesis, where the expression of numerous genes is regulated in a spatiotemporally specific manner^8^. Moreover, miRNAs regulate epithelial tissue homeostasis in adults by modulating genes involved in the (i) maintenance of long term and self-renewal and differentiation capabilities of undifferentiated stem cells (ii) dynamics/interactions between undifferentiated stem cells and epithelial cells^8^. miRNAs such as miR-17, miR-29, miR-31, miR-124 and miR-200, have been reported to regulate multiple cell adhesion genes^5^. Moreover, hsa-miR-1249-3p has been reported to regulate numerous pathways in carcinoma cells such as proliferation, adhesion, migration, survival, and apoptosis. This miRNA can also control the epithelial-mesenchymal transition (EMT), whereby epithelial cells lose cell polarity and cell–cell adhesion potentials, and gain migratory properties^9,10^. Therefore, hsa-miR-1249-3p results as an attractive candidate in understanding the regulation of human epithelial cell activities and adhesion mechanisms.

The purpose of this study was to investigate the role of hsa-miR-1249-3p on epithelial cell functions and explore the possible underlying mechanisms. In vitro and ex vivo hsa-miR-1249-3p expression was investigated in both epithelial cells and tissues. Then, the hsa-miR-1249-3p target gene Homeobox A13 (HOXA13) and its downstream cell adhesion gene β-catenin, were studied^11,12^*.* Indeed, β-catenin is a critical component of E-cadherin cell adhesion complexes which control the epithelial tissue architectural integrity, while the protein also play a key role in the WNT/β-catenin pathway^13^. In addition, epithelial cell processes, such as colony forming potential, proliferation, migration and apoptosis^14^ were also evaluated herein. Hsa-miR-1249-3p was upregulated in human epithelial cells and uterine cervical tissues, compared to cervical carcinoma cells and precancerous tissues, respectively. Hsa-miR-1249-3p inhibition prompted the mRNA and protein upregulation of HOXA13 which, in turn, favored the protein expression of β-catenin in epithelial cells. Moreover, a strong colony forming effect was determined in hsa-miR-1249-3p-inhibited epithelial cells.

## Results

### Hsa-miR-1249-3p is overexpressed in epithelial cell lines and tissues

**Figure 1:**
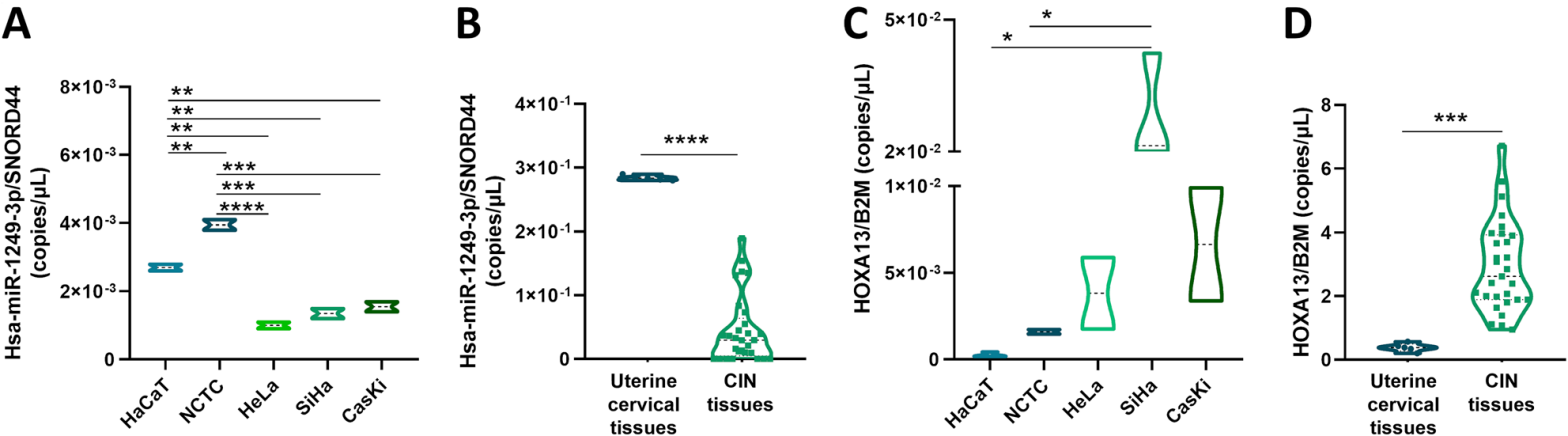
Hsa-miR-1249-3p and HOXA13 mRNA expression levels in human epithelial and cervical carcinoma cell lines, as well as in uterine cervical tissues and precancerous lesions. Experiments were performed on two biological replicates of epithelial cell lines, i.e. HaCaT and NCTC, and three biological replicates of cervical cancer cell lines, i.e. HeLa, SiHa and CasKi, as well as on all normal/CIN tissue specimens. Three technical replicates per each cell line/tissue sample were used for each ddPCR experiment. (**A**) Hsa-miR-1249-3p was overexpressed in epithelial cell lines i.e. NCTC, HaCaT compared to cervical carcinoma cells i.e. HeLa SiHA and CasKi. (**B**) Hsa-miR-1249-3p was overexpressed in cervical uterine tissues (n = 5) compared to Cervical intraepithelial neoplasia (CIN, n = 30) tissues. (**C**) HOXA13 mRNA was overexpressed in epithelial cell lines, i.e. NCTC, HaCaT compared to cervical carcinoma cells, i.e. HeLa SiHA and CasKi. (**D**) HOXA13 mRNA expression levels were downexpressed in uterine cervical tissues (n = 5) compared to CIN tissues (n = 30). Data were reported as copies/μL of hsa-miR-1249-3p and HOXA13 normalized over the value of SNORD44 (copies/μL) and B2M (copies/μL) over the value of SNORD44 (copies/μL) and B2M (copies/μL) housekeeping genes, respectively. Values were analyzed using the D’Agostino Pearson normality test. Student t test or one-way analysis of variance (ANOVA test) were then used for normal distributions, while Mann–Whitney *U* or Kruskal–Wallis tests were used for non-normal distributions. All panels: *p < 0.05, **p < 0.01, ***p < 0.001 and ****p < 0.001.

Since hsa-miR-1249-3p has been previously described as dysregulated in various carcinoma types^9,10^, we have evaluated its expression in normal epithelial cells in comparison with uterine cervical carcinoma cell lines. Hsa-miR-1249-3p expression levels were quantitatively evaluated by ddPCR in human epithelial cells HaCaT and NCTC and compared to those evaluated in uterine cervical cancer cell lines HeLa, CasKi and SiHa. HaCaT and NCTC cell lines revealed hsa-miR-1249-3p levels of 2.7 × 10^–3^ (± 1.4 × 10^–4^) and 4 × 10^–3^ (± 2.1 × 10^–4^), respectively, while cervical cancer cell lines HeLa, SiHa and CasKi showed hsa-miR-1249-3p levels of 1 × 10^–3^ (± 1.41 × 10^–4^), 1.4 × 10^–3^ (± 2.2 × 10^–4^) and 1.6 × 10^–3^ (± 2.1 × 10^–4^), respectively. In HaCaT and NCTC cell lines hsa-miR-1249-3p levels were significantly higher than in each HeLa, SiHa and CasKi cell line (p < 0.01; Fig. 1, panel A). To confirm the quantitative data obtained in vitro, hsa-miR-1249-3p expression levels were evaluated ex vivo in uterine cervical tissues (n = 5) and compared to those found in precancerous CIN (n = 30) tissues. Hsa-miR-1249-3p was detectable in all uterine cervical tissues and in 24/30 (80%) CIN tissues. The hsa-miR-1249-3p levels were 2.8 × 10^–1^ (± 4 × 10^–3^) and 0.5 × 10^–1^ (± 5.3 × 10^–2^) in uterine cervical and CIN tissues, respectively. Hsa-miR-1249-3p levels were significantly higher in uterine cervical tissues than that of CIN tissues (p < 0.0001, Fig. 1, panel B).

### HOXA13 mRNA is downregulated in epithelial cell lines and uterine cervical tissues

HOXA13 has been previously identified experimentally as a direct and functional target of hsa-miR-1249-3p^12^. As hsa-miR-1249-3p resulted herein as differentially expressed across epithelial and cancer cell lines and CIN tissues, the expression levels of HOXA13 transcript were afterwards evaluated in human epithelial cell lines HaCaT and NCTC and in cervical cancer cell lines HeLa, SiHa and CasKi by ddPCR, as well as in uterine cervical (n = 5) and CIN (n = 30) tissues.

HaCaT and NCTC cells revealed HOXA13 mRNA levels of 2.7 × 10^–4^ (± 1.5 × 10^–4^) and 1.6 × 10^–3^ (± 2 × 10^–4^), respectively. Cervical cancer cell lines HeLa, SiHa and CasKi showed HOXA13 mRNA levels of 3.8 × 10^–3^ (± 3 × 10^–3^), 2.8 × 10^–2^ (± 1.3 × 10^–2^) and 6.6 × 10^–3^ (± 4.6 × 10^–3^), respectively. In NCTC and HaCaT cell lines, HOXA13 mRNA levels were significantly lower compared to SiHa cells (p < 0.05; Fig. 1, panel C). In uterine cervical and CIN tissues, HOXA13 mRNA levels were 3.9 × 10^–1^ (± 1.3 × 10^–1^) and 2.93 × 10^0^ (± 1.4), respectively. HOXA13 mRNA levels resulted as significantly lower in uterine cervical tissues than in CIN tissues (p < 0.001, Fig. 1, panel D). These results suggest that HOXA13 mRNA expression is downregulated in epithelial cells and tissues. Spearman correlation analysis was performed to evaluate a possible association between hsa-miR-1249-3p and HOXA13 mRNA levels in epithelial and cancer cell lines and pre-cancerous tissues. Correlation analysis performed in HaCaT, NCTC, HeLa, SiHa and CasKi revealed the presence of a slight, but not significant, inverse correlation, with a Spearman coefficient r of − 0.70 (p > 0.05). CIN tissues indicated a slight, and not significant, inverse correlation, with a Spearman coefficient r of − 0.214 (p > 0.05).

### HOXA13 mRNA expression is modulated by hsa-miR-1249-3p in human epithelial cells

**Figure 2:**
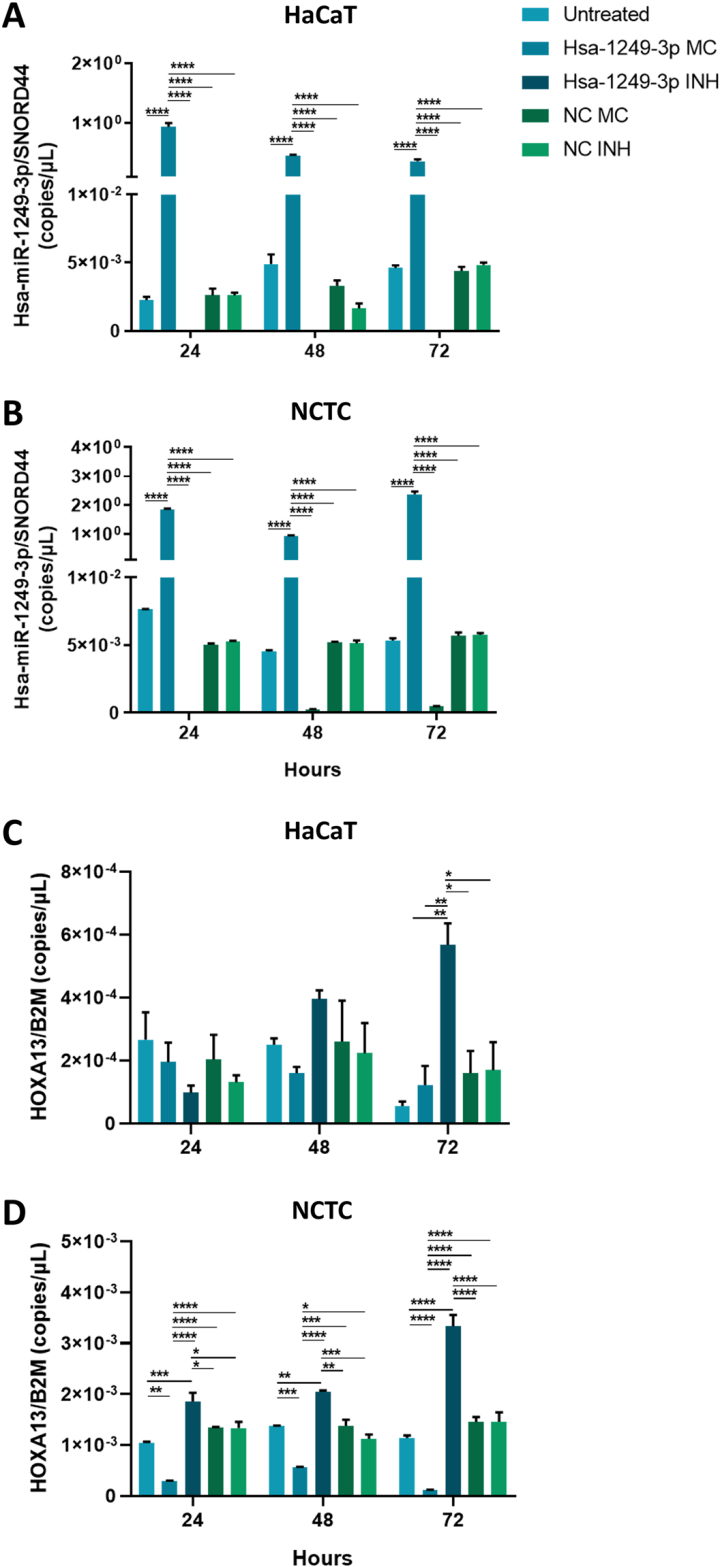
Quantification of hsa-miR-1249-3p and HOXA13 and β-catenin expression levels in hsa-miR-1249-3p mimic, hsa-miR-1249-3p inhibitor, and negative controls of transfection in HaCaT and NCTC cells. (**A**,**C**) Hsa-miR-1249-3p and HOXA13 mRNA levels in HaCaT cell line at 24, 48 and 72 h after transfection with miR-mi, miR-inh and mimic/inhibitor negative controls (NCs). (**B**,**D**) Hsa-miR-1249-3p and HOXA13 mRNA levels in NCTC cells at 24, 48 and 72 h after transfection with miR-mi, miR-inh and NCs. Experiments were performed on two biological replicates, i.e. HaCaT and NCTC cells, of which three technical replicates per experimental condition were used. Data were reported as copies/μL of hsa-miR-1249-3p and HOXA13 normalized over the value of SNORD44 (copies/μL) and B2M (copies/μL) housekeeping genes, respectively. All panels: *MC* mimic, *INH* inhibitor, *miR*-*mi* hsa-microRNA-1249-3p-mimic, *miR*-*inh* hsa-microRNA-1249-3p-inhibitor; *p < 0.05, **p < 0.01, ***p < 0.001 and ****p < 0.001. Values were analyzed using the D’Agostino Pearson normality test. One/two-way analysis of variance (ANOVA test) were then used for normal distributions, while Kruskal–Wallis test was used for non-normal distributions.

We next evaluated whether HOXA13 is regulated by hsa-miR-1249-3p in HaCaT and NCTC cells. Cells were transfected with hsa-miR-1249-3p mimic, hsa-miR-1249-3p inhibitor, and their corresponding negative controls. Upon transfections, hsa-miR-1249-3p resulted as undetectable in miR-inhibitor HaCaT condition, at 24, 48 and 72 h. Consistently, the miRNA resulted strongly overexpressed in miR-mimic HaCaT cells, with hsa-miR-1249-3p levels of 9.3 × 10^–1^ (± 6.4 × 10^–2^), 4.6 × 10^–1^ (± 1.5 × 10^–2^) and 3.6 × 10^–1^ (± 2.8 × 10^–2^) at 24, 48 and 72 h, respectively, compared to untreated cells and negative controls (p < 0.0001, Fig. 2, panel A). Similarly, hsa-miR-1249-3p resulted to be almost undetectable in miR-inhibitor NCTC condition, at 24, 48 and 72 h. At the same time, hsa-miR-1249-3p was strongly overexpressed in miR-mimic NCTC cells, whose levels resulted to be 1.86 (± 2.6 × 10^–2^), 0.94 (± 1.7 × 10^–2^) and 2.4 (± 9.7 × 10^–2^) at 24, 48 and 72 h, respectively, compared to untreated cells and negative controls (p < 0.0001, Fig. 2, panel B). Then, HOXA13 mRNA levels were evaluated in miR-inhibitor and miR-mimic HaCaT and NCTC cells and their corresponding negative controls. At 72 h upon transfections, a significant overexpression of HOXA13 mRNA, whose levels were estimated as 5.7 × 10^–4^ (± 6.8 × 10^–5^), was found in miR-inhibitor HaCaT condition compared to untreated cells, 1.2 × 10^–4^ (± 6.1 × 10^–5^), miR-mimic HaCaT cells, 1.2 × 10^–4^ (± 6.2 × 10^–5^) and negative controls, 1.6–1.7 × 10^–4^ (± 7.1–8.9 × 10^–5^) (p < 0.05, Fig. 2, panel C). HOXA13 mRNA levels were similar across the experimental conditions at 24 and 48 h, despite a slight, but not significant, increment was visible from 24 to 72 h in the miR-inhibitor HaCaT condition (p > 0.05, Fig. 2, panel B). HOXA13 mRNA levels resulted significantly upregulated in miR-inhibitor NCTC condition, whose levels resulted to be 1.8 × 10^–3^ (± 1.7 × 10^–4^), 2.1 × 10^–3^ (± 2 × 10^–5^) and 3.3 × 10^–3^ (± 2.2 × 10^–4^) at 24, 48 and 72 h, respectively, compared to untreated cells and negative controls (p < 0.05, Fig. 2, panel D). At the same time, HOXA13 mRNA levels were lower in miR-mimic NCTC cells, whose levels resulted to be 2.9 × 10^–4^ (± 6.9 × 10^–6^), 5.7 × 10^–4^ (± 4 × 10^–6^) and 1.2 × 10^–4^ (± 2.8 × 10^–6^) at 24, 48 and 72 h, respectively, compared to untreated cells and negative controls (p < 0.05, Fig. 2, panel D).

### Hsa-miR-1249-3p inhibition in human epithelial cells overexpresses HOXA13 protein and its downstream cell adhesion protein β-catenin

**Figure 3:**
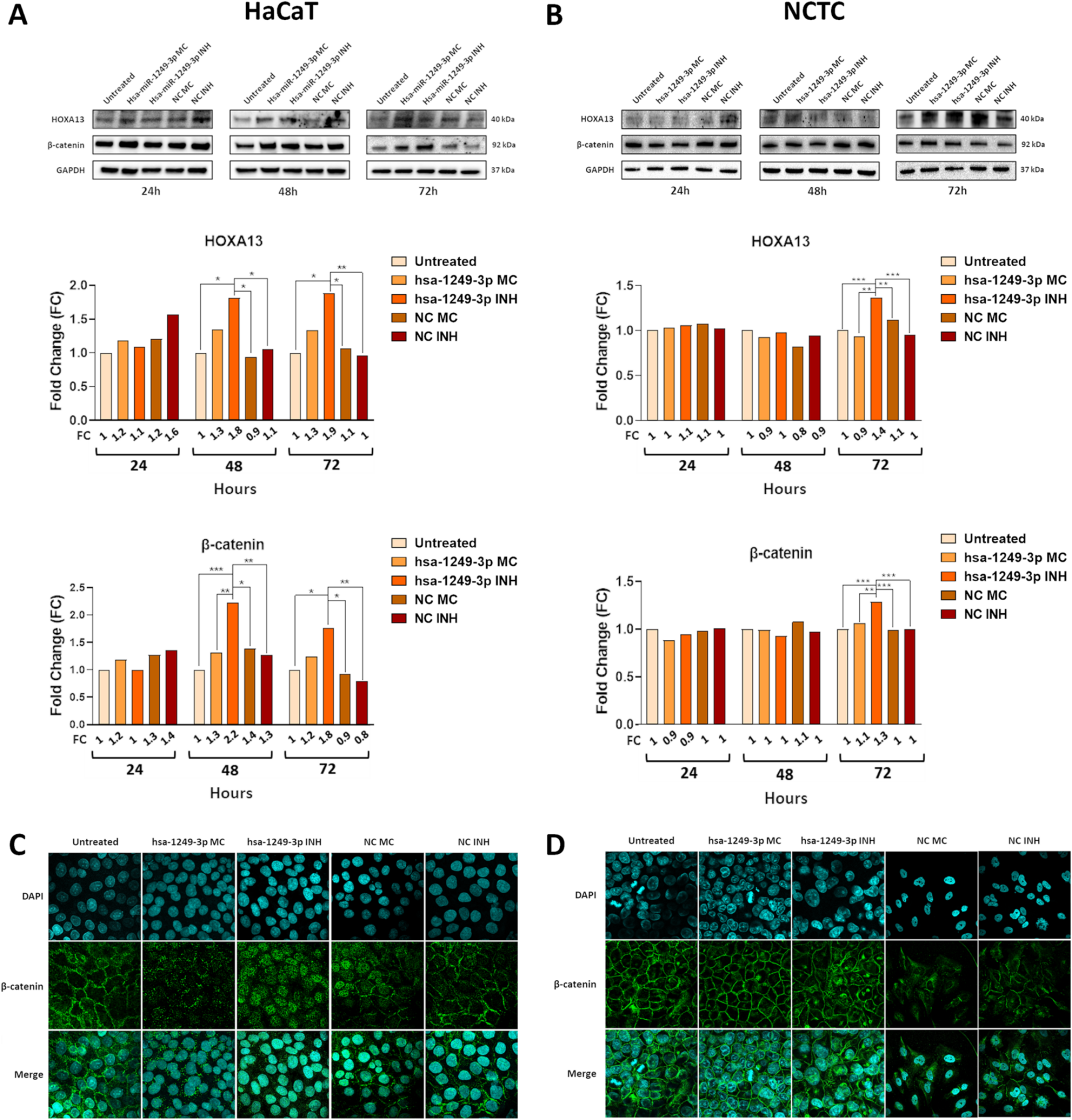
Quantification of HOXA13 and β-catenin protein expression levels in hsa-miR-1249-3p mimic, hsa-miR-1249-3p inhibitor, and negative controls of transfection and localization of β-catenin by immunofluorescent staining in HaCaT and NCTC cells. (**A**,**B**) Western blot (WB) data were analyzed by densitometric quantification of HOXA13 (40 kDa) and β-catenin (92 kDa) protein levels in HaCaT and NCTC cell lines at 24, 48 and 72 h after transfection with miR-mi, miR-inh and mimic/inhibitor negative controls (NCs). Protein levels were normalized to GAPDH (37 kDa). Results are shown as relative Fold Change (FC). (**C**,**D**) Experiments were conducted in miR-mi, miR-inh, and negative controls of transfection conditions. Confocal microscopic images of HaCaT and NCTC cells stained with FITC tagged anti-β-catenin antibody (green), DAPI for nucleus (blue). Images were acquired with an Olympus FV3000 confocal microscope equipped with a 60 × oil-immersion objective and processed by using the open-source Fiji software. Merge: overlay images of anti-β-catenin and DAPI. All panels: *MC* mimic, *INH* inhibitor, *miR*-*mi* hsa-microRNA-1249-3p-mimic, *miR*-*inh* hsa-microRNA-1249-3p-inhibitor; *p < 0.05, **p < 0.01, ***p < 0.001. Membranes were cut prior to hybridization with antibodies. In order to improve the representation of the panels, original blot images were cropped. Original blots with multiple exposure images at different times with membrane edges visible were included in Supplementary File 2. Values were analyzed using the D’Agostino Pearson normality test. One/two-way analysis of variance (ANOVA test) were then used for normal distributions, while Kruskal–Wallis test was used for non-normal distributions.

To further clarify the role of hsa-miR-1249-3p in epithelial cells, the expression of HOXA13 and β-catenin, which plays a role in the epithelial cell adhesion^15^, were examined by WB analysis in HaCaT and NCTC cells. Upon transfections, HOXA13 protein expression was found to be increased in miR-inhibited HaCaT cells of 1.8-, 1.9- and 1.7-fold compared to untreated, NC mimic and NC inhibitor cells at 48 h, respectively (p < 0.05, Fig. 3, panel A). A similar expression pattern was detected at 72 h, showing a 1.9-fold increase in HOXA13 protein levels in miR-inhibitor HaCaT cells compared to untreated cells. At the same time, the HOXA13 protein levels were 1.8- and 1.9-fold higher in miR-inhibited HaCaT cells compared to NC mimic and NC inhibitor cells, respectively (p < 0.05, Fig. 3, panel A). In NCTC cells, miR-inhibitor condition showed an increase in HOXA13 protein levels by 1.4-, 1.5-, 1.3- and 1.4-fold compared to untreated, miR-mimic, NC mimic and NC inhibitor cells, respectively, at 72 h (p < 0.01, Fig. 3, panel B).

β-catenin protein expression was evaluated in miR-inhibitor and miR-mimic HaCaT and NCTC cell lines and their corresponding NCs at 24, 48 and 72 h. At 48 h, a 2.2- and 1.9-fold increase in β-catenin protein levels was detectable in miR-inhibitor HaCaT cells compared to untreated and miR-mimic cells, respectively, while a 1.8- and 1.9-fold increase in β-catenin protein levels was found in miR-inhibited cells compared to NC mimic and NC inhibitor cells, respectively (p < 0.05, Fig. 3, panel A). At 72 h, the β-catenin protein levels were 1.8-fold higher in miR-inhibitor HaCaT cells compared to untreated, and 1.9- and twofold higher in miR-inhibited cells compared to NC mimic and NC inhibitor control cells, respectively (p < 0.05, Fig. 3, panel A). In NCTC cells, miR-inhibitor condition showed an increase in HOXA13 protein levels of 1.3-, 1.2-, 1.3- and 1.3-fold compared to untreated, miR-mimic, NC mimic and NC inhibitor controls, respectively, at 72 h (p < 0.01, Fig. 3, panel B).

### Localization of β-catenin in human epithelial cells

HOXA13 can bind to β-catenin and promote the nuclear accumulation of β-catenin, as demonstrated before^11^. As shown in Fig. 3, miR-inhibitor and miR-mimic HaCaT and NCTC cells and their corresponding negative controls, were evaluated for the cellular localization of β-catenin. Confocal immunofluorescence staining indicated that β-catenin protein localized into the nucleus of hsa-miR-1249-3p inhibited HaCaT cells, as shown by the overlapping between DAPI stain and β-catenin green fluorescence. A slight nuclear localization of β-catenin was also determined in NCTC cells, as DAPI stain overlapped with β-catenin green fluorescence in several nuclear locations. Contrariwise, β-catenin protein was found frequently associated to the cell cytoplasm/membrane in miR-mimic HaCaT and NCTC cells and negative controls (Fig. 3, panel C and D).

### Hsa-miR-1249-3p inhibition is associated with changes in colony forming potential of human epithelial cells

**Figure 4:**
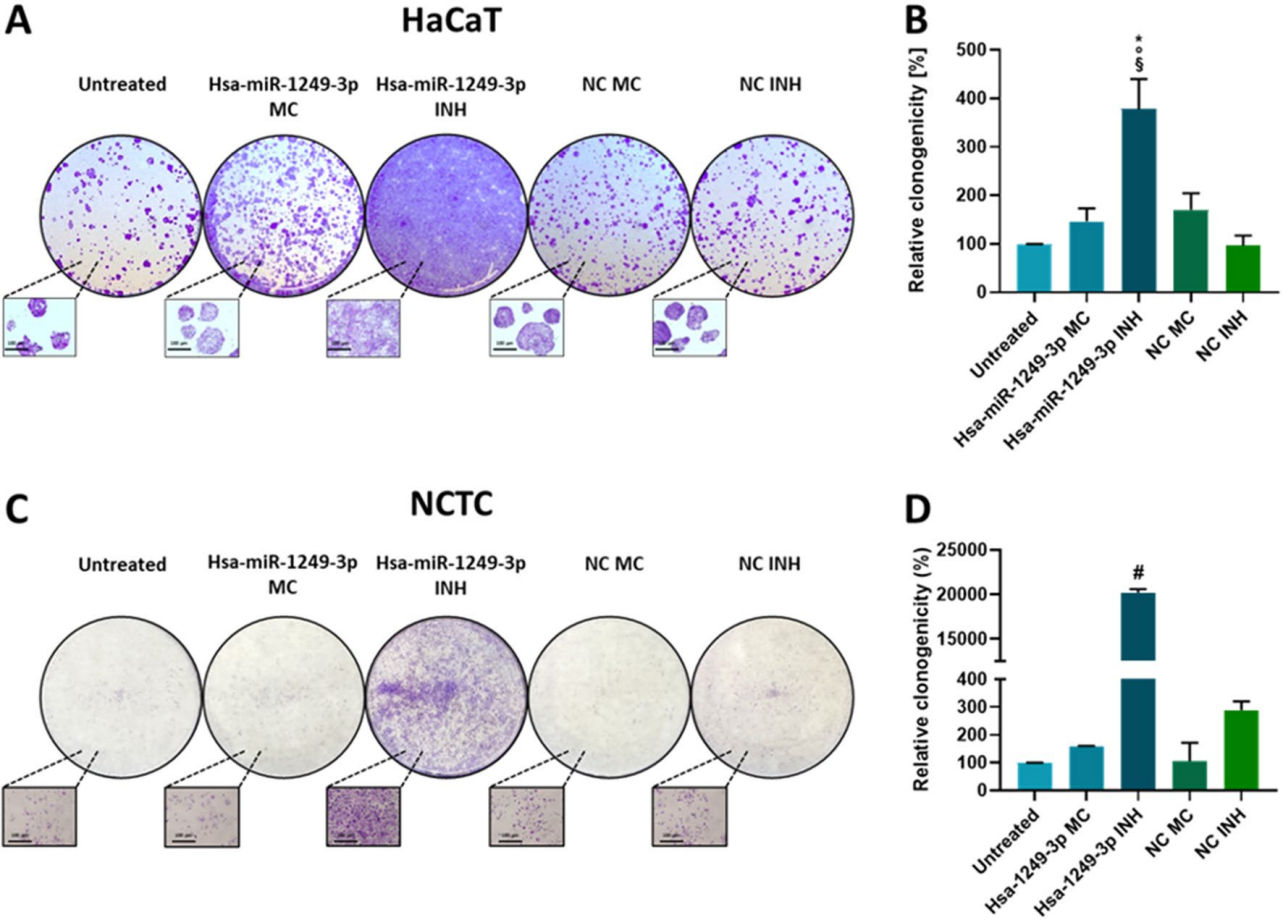
HaCaT and NCTC cell colony formation evaluation in hsa-miR-1249-3p mimic, hsa-miR-1249-3p inhibitor, and negative controls of transfection. (**A**,**C**) Cells were stained with 0.5% crystal violet dye after 72 h of transfections. (**B**,**D**) Graphical data represent the level of colony formation after transfection compared to untreated control values. Values are presented as the mean % ± standard error of mean (SEM). (**B**) ^§^p < 0.001, miR-inh vs untreated, °p < 0.01, miR-inh vs miR-mi and vs NC inhibitor; *p < 0.05 miR-inh vs NC mimic. (**D**) ^#^p < 0.0001, miR-inh vs untreated, vs miR-mi, vs NC mimic and vs NC inhibitor. All panels: *MC* mimic, *INH* inhibitor, *miR*-*mi* hsa-microRNA-1249-3p-mimic, *miR*-*inh* hsa-microRNA-1249-3p-inhibitor. Experiments were performed on two biological replicates, i.e. HaCaT and NCTC cells, of which three technical replicates per experimental condition were used. Values were analyzed using the D’Agostino Pearson normality test. One -way analysis of variance (ANOVA test) was then used for normal distributions, while Kruskal–Wallis test was used for non-normal distributions.

HOXA13 has previously been demonstrated to regulate cell adhesion capability^16^ and EMT^17,18^. Moreover, hsa-miR-1249-3p expression has been reported to counteract EMT^9^. Given the regulatory effect of hsa-miR-1249-3p/HOXA13 axis in β-catenin gene expression in HaCaT and, in less extend, in NCTC cells, we evaluated the implication in regulating the cell adhesion by cell colony forming assay^13,19^. As shown in Fig. 4, miR-inhibitor and miR-mimic HaCaT cells and their corresponding negative controls, were evaluated for their colony forming potential by colony forming assay, at 72 h. Upon transfections, a significant increase of the cell colony forming potential was observed in miR-inhibitor HaCaT condition (378%, ± 62.4) compared to untreated (99.3%, ± 0.5) and miR-mimic cells (146.3%, ± 27.1) (p < 0.001, miR-inhibitor HaCaT *vs* untreated; p < 0.01, miR-inhibitor HaCaT *vs* miR-mimic cells, Fig. 4, panels A and B). Similarly, a significantly strong increase of the cell colony forming potential was determined in miR-inhibitor NCTC condition (20,214%, ± 545) compared to untreated (99% ± 1.4) and miR-mimic cells (159%, ± 2) (p < 0.00001, Fig. 4, panels C and D). In addition, the cell colony forming potential in miR-inhibitor NCTC condition was higher compared to both NC-mimic (106% ± 93) and NC-inhibitor (289%, ± 44) cells (p < 0.0001, Fig. 4, panels C and D).

### Hsa-miR-1249-3p is not implicated in human epithelial proliferation, migration and apoptosis

The possible implication of hsa-miR-1249-3p/HOXA13 axis in regulating epithelial cell proliferation, migration and apoptosis was investigated. Cell proliferation, migration and apoptosis were evaluated in miR-inhibitor and miR-mimic HaCaT cells and their corresponding negative controls, at 24, 48 and 72 h. Upon transfections, both cell proliferation and migration capabilities in HaCaT cells were not significantly altered in any experimental condition (p > 0.05, Supplementary File 1). Similarly, NCTC cell migratory potential was not altered in any experimental condition (p > 0.05, Supplementary File 1). The expression of anti-/pro-apoptotic protein markers PARP-1, BCL-XL and caspase-3 resulted similar among the experimental conditions at 24, 48 and 72 h (Supplementary File 1).

## Discussion

This study aimed to investigate the role of hsa-miR-1249-3p in human epithelial cell functions and mechanisms. Hsa-miR-1249-3p resulted as overexpressed in human epithelial cells/cervical tissues compared to cervical carcinoma cells/precancerous tissues, with the opposite expression of its validated target gene HOXA13. Functional experiments indicated that hsa-miR-1249-3p regulates the expression of HOXA13 and its downstream cell adhesion gene β-catenin in epithelial cells and that the modulation of this miRNA is associated with changes in the epithelial cell colony forming potential.

The mechanisms relying on the epithelial cell adhesion have gradually been uncovered. miRNAs are acquiring importance, as being capable of targeting cell adhesion genes, whilst impairments at this regulatory level can lead to diseases^20^, such as epithelial tumors^5^. The miRNA role on epithelial cell adhesion was therefore given great interest herein. In this study, hsa-miR-1249-3p was found to be overexpressed in epithelial cells compared to cervical carcinoma cells; further analyses conducted on uterine cervical and precancerous lesion tissues confirmed these data. These findings suggest that hsa-miR-1249-3p may play a biological role in epithelial cells. A physiological regulatory role for miR-1249 has been described during osteogenic differentiation^21,22^, while its impaired expression has been documented in cancer^10,23,24^, and in other non-tumor diseases/conditions^25–34^. In cancer, both tumor suppressor and oncogenic features have been described for hsa-miR-1249-3p^12,35–38^. Despite hsa-miR-1249-3p activity affects different carcinoma cell processes such as proliferation, adhesion, migration, survival, EMT and apoptosis, its functional and biological role in epithelial cells remains to be elucidated.

miRNAs exert their regulatory activity on gene expression by targeting genes involved in multiple pathways. Hsa-miR-1249-3p has previously been reported to regulate HOXA13 gene in lung cancer cells^12^. HOXA13 is a gene known to be dysregulated in carcinomas^39^. Herein, the downregulation of HOXA13 mRNA, paralleled with the hsa-miR-1249-3p overexpression, was found in epithelial cells and cervical tissues. These expression patterns were partially confirmed by Spearman analysis, as an inverse correlation seemed to be present although being not significant, probably as a consequence of the reduced number of samples available.

In this study, the hsa-miR-1249-3p/HOXA13 interplay was functionally demonstrated in epithelial cells, as HOXA13 overexpression was detectable in miR-inhibited condition. This result indicates that hsa-miR-1249-3p modulates HOXA13 in epithelial cells, confirming and extending previous findings^12^. Contrariwise, the lack of modulation of HOXA13 in hsa-miR-1249-3p mimic condition, which was determined in HaCaT cells but not in NCTC, might be due to the low endogenous expression of HOXA13 determined in HaCaT cells. HOXA13 belongs to the transcription factor family of homeobox genes involved in the embryo development. As HOXA13 has been reported as dysregulated in numerous carcinoma types^11,18,39–43^, an oncogenic role for this gene has been described^18,44,45^. Moreover, its forced overexpression in esophageal keratinocytes conferred oncogenic features to these cells^46^. Considering these aspects, an implication of HOXA13 in regulating epithelial cell functions might be plausible. Herein, the hsa-miR-1249-3p/HOXA13 axis was functionally explored in modulating the cell colony forming process^47^. Notably, a strong colony forming effect, paralleled with a slight, but not significant, proliferation increase, was found in miR-inhibited epithelial cells overexpressing HOXA13. This evidence points in favor towards a possible role of hsa-miR-1249-3p/HOXA13 axis in this cell process.

To provide insights into the underlying mechanism, we evaluated the relationship between hsa-miR-1249-3p/HOXA13 axis and the cell adhesion coordinator β-catenin^15^, whose expression has been reported to be under HOXA13 regulation^11,48^, and to control the cell colony forming potential^19^. Notably, β-catenin protein was increased in miR-inhibited epithelial cells expressing HOXA13. Moreover, confocal immunofluorescence staining indicated β-catenin protein distribution into the nucleus in hsa-miR-1249-3p inhibited HaCaT cells and, to a lesser extent, NCTC cells, as opposed to control conditions, in which the protein was found frequently confined to the cell cytoplasm/membrane. This result corroborates the hypothesis that hsa-miR-1249-3p regulates HOXA13 and, in turn, its downstream gene β-catenin. HOXA13 might bind to β-catenin in epithelial cells and promote, in turn, the nuclear accumulation of β-catenin, as previously demonstrated in colon cancer cells^11^. Indeed, β-catenin typically shows a plasma membrane/cell cytoplasm localization in HaCaT cells^49^. It should be underlined that β-catenin exerts a key role in cell adhesion, as being a component of the cadherin-based adherens junctions, which control the structural integrity and functional polarization of epithelia^4,50^. β-catenin particularly anchors the intracellular domain of the transmembrane protein cadherin facilitating its connection with the cytoskeleton. In physiological conditions, the subcellular amount of membrane-associated β-catenin dynamically controls the epithelial cell adhesion^4^. On this ground, β-catenin might be under hsa-miR-1249-3p/HOXA13 axis regulation to control the integrity of epithelial adherens junctions. β-catenin is also the core component of the WNT/β-catenin pathway, which regulates the embryo development/adult tissue homeostasis and genes involved in cell proliferation, migration, and differentiation^51^. WNT/β-catenin activation can promote the accumulation of β-catenin in the nucleus of transformed cells^52^. In addition, WNT/β-catenin pathway dysregulation can promote carcinogenesis via EMT induction^15^, through which epithelial cells lose their polarity and adhesion capacities and acquire mesenchymal features^53^. In this context, an involvement of hsa-miR-1249-3p/HOXA13 axis in EMT cannot be excluded. As a support, the forced hsa-miR-1249-3p expression has been reported to reverse EMT and suppress proliferation/migration abilities in breast carcinoma cells^9^. WNT/β-catenin pathway is also under HOXA13 regulation in colon cancer, leading tumor formation promotion^11^. The modulation of β-catenin and nuclear localization showed herein alongside the cell colony forming potential and the lack of effects on cell migration and apoptosis, suggest that hsa-miR-1249-3p/HOXA13 axis may play a role in the epithelial cell adhesion, and possibly EMT. Indeed, the β-catenin knockdown has been reported to decrease the clonogenicity of colorectal carcinoma cells^19^, while a connection between decreased EMT and cell clonogenicity and migration inhibition has been reported in breast carcinoma cells^54^. Evaluating the EMT markers such as epithelial markers cytokeratins and E-cadherin and mesenchymal markers such as N-cadherin, vimentin, and fibronectin, in epithelial cells in relation to hsa-miR-1249-3p should be further considered. At the same time, attachment assays aimed in evaluating cell proliferation, morphology change, and attachment quality would allow to obtain a more comprehensive overview of the regulative role of hsa-miR-1249-3p/HOXA13 axis upon epithelial cell functions. These experiments are feasible and can be further performed.

The main limitation of the study is the limited number of cell lines employed for functional experiments. Indeed, the activity of hsa-miR-1249-3p/HOXA13 axis may be multifaceted across different epithelial cell lines. The regulatory role of hsa-miR-1249-3p/HOXA13 axis on epithelial cells needs to be further evaluated in additional epithelial cell lines.

## Conclusions

In conclusion, this study provides novel insights on the miRNA-based mechanisms regulating epithelial cell functions. The modulation of hsa-miR-1249-3p is associated with changes in the potential of epithelial cells to form colonies, while this miRNA can regulate the expression of HOXA13 and, in turn, its downstream cell adhesion gene β-catenin. These data will allow the set-up of further studies aimed in exploring the relationship between the hsa-miR-1249-3p/HOXA13 axis and downstream cell adhesion genes whose dysregulations may be involved in EMT and cell transformation processes. Moreover, our data will allow to further investigate the implication of candidate upstream regulators of hsa-miR-1249-3p/HOXA13 axis on epithelial cell functions, such as the long non-coding RNA MIF-AS1, whose regulatory activity on hsa-miR-1249-3p has been demonstrated in cancer cells^9^. Evaluating the upstream regulators of hsa-miR-1249-3p/HOXA13 axis in epithelial cells would shield novel light into the miRNA-based mechanisms regulating epithelial cell functions.

## Materials and methods

### Cell lines and tissues

Human epithelial cell lines HaCaT and NCTC and cervical cancer cell lines HeLa, SiHa and CasKi were cultured in DMEM F12 medium with 10% fetal bovine serum (FBS, EuroClone) and 1% pen/strep at 37 °C and 5% CO_2_. Normal epithelial (n = 5) and histologically confirmed precancerous cervical intraepithelial neoplasia (CIN, n = 30) formalin and fixed paraffin and embedded tissue specimens (FFPE), obtained from diagnostic biopsies, were provided by the Pathology Unit, University Hospital of Ferrara, Ferrara, Italy. Institutional Review Board approval was obtained from University Ferrara Hospital Ethical Committee (ID:160986). Informed written consents were obtained from patients/subjects. The study was performed in accordance with the Declaration of Helsinki (2008).

### Transfections

HaCaT and NCTC cell lines were transfected with mirVana™ miRNA mimic, hsa-miR-1249-3p (Invitrogen™), mirVana™ miRNA inhibitor, hsa-miR-1249-3p (Invitrogen™) as well as with negative/positive controls (NCs) (Invitrogen™) at 50 nM using Lipofectamine™ RNAiMAX Transfection Reagent (Invitrogen™) in Opti-MEM Reduced Serum Medium, no phenol red (Gibco™)^55^. Untreated HaCaT cells were used as control. Transfections were performed 24 h after cell seeding. miRNA mimic, inhibitor, and NCs were administered on the first day of treatment. Molecular/phenotypical effects of transfections were measured at 24, 48 and 72 h after transfection^14^.

### Cell colony forming, proliferation and migration assays

Cell colony forming, proliferative and migration abilities were evaluated in treated and untreated cells with hsa-miR-1249-3p mimic and inhibitor, along with NCs at 24, 48 and 72 h after transfection. HaCaT and NCTC cell colony forming potential was evaluated by colony forming assay by seeding 10^4^ cells/well for each experimental condition in six-well plates, in duplicate. After 72 h of incubation, cells were washed with phosphate buffered saline (PBS) and fixed with cold methanol. Lastly, cells were stained with 0.5% crystal violet dye for 10 min. Colonies containing > 50 cells were counted for each experimental condition and result expressed as Colony Formation Efficiency^56^. HaCaT cell proliferation was assessed using the WST-1 assay in 96-well plates (Roche, Milan, Italy). A total of 6 × 10^3^ cells/well were seeded for each experimental condition. Cell proliferation was calculated as [(optical density value/baseline value) × 100], where baseline values corresponds to the values of untreated cells. HaCaT cell migration (wound healing) assay was performed to evaluate the migration ability of cells to close a wound. Untransfected cells were seeded at density of 3 × 10^5^ cells/well in 24-well plates, in duplicate. After reaching a > 90% of confluence, cells were washed with PBS, independently treated with hsa-miR-1249-3p mimic, inhibitor and with negative/positive controls and incubated for 48 h at 37 °C and 5% CO_2_. After this period, a linear scratch was performed in the cell monolayer of each well. Cells were then incubated for 72 h, replacing medium every 24 h. The wound was firstly evaluated to assess the starting area and then examined every 24 h. Cell migration was measured by comparing the healed area of the scratch among the different experimental conditions. Results were expressed as percentage of cell migration in each check point [(Area t0h—Area txh)/(Area t0h)] × 100). Colony formation and migration measurements were performed with ImageJ software.

### RNA isolation, cDNA preparation and droplet-digital PCR

Total RNA was isolated from cells and tissues, using the miRNeasy Mini Kit (Qiagen, Milan Italy) and QIAamp RNeasy FFPE (Qiagen, Milan Italy), respectively. RNAs were quantified spectrophotometrically with the NanoDrop 2000 (Thermo, Milan, Italy) and stored at − 80 °C until analyses. miRNAs were retro-transcribed using the miRCURY LNA RT kit (Qiagen, Milan Italy). Total mRNA was retro-transcribed using ImProm-II™ reverse RT. Products were stored at − 20 °C until analysis. Hsa-miR-1249-3p and HOXA13 mRNA expressions were evaluated by droplet-digital (ddPCR), with the QX200ddPCR system (Bio-Rad, Segrate, Italy). DdPCR allows the absolute quantification of nucleic acid molecules in a biological/clinical sample, without requiring data normalization with the housekeeping gene^57^. The ddPCR Supermix for Probes (no dUTP) (Bio-Rad, Segrate, Italy) was used in combination with the TaqMan™ Gene Expression Assay (FAM) for HOXA13 (Thermo, Milan, Italy). Assay ID is Hs04194761_s1 (Thermo, Milan, Italy). Upon mix preparation, an emulsion was produced in the automated droplet generator (Bio-Rad, Segrate, Italy). DdPCR plate was then heat-sealed with foil and placed in the SimpliAmp Thermal Cycler (Thermo Fisher Scientific, Milan, Italy). Plate reading was done with the Qx 200 Droplet Reader (Bio-Rad, Segrate, Italy). SNORD44 and B2M were employed as housekeeping genes to normalize the amount of hsa-miR-1249-3p and HOXA13 mRNA, respectively. Expression data were reported as copies/μL of hsa-miR-1249-3p and HOXA13 normalized over the value of SNORD44 and B2M housekeeping genes (copies/μL), respectively. Amplification conditions were as follows: (i) hsa-miR-1249-3p and SNORD44, 5 min of enzyme activation at 95 °C, followed by 40 cycles of 30 s at 94 °C, 1 min at 56 °C and by 5 min at 4 °C and 5 min at 90 °C^58^; (ii) HOXA13 and B2M, 10 min of enzyme activation at 95 °C, followed by 40 cycles of 30 s at 94 °C, 1 min at 55 °C and 10 min at 98 °C. When appropriate, results are reported as mean miRNA and/or mRNA levels ± standard deviation of mean (SD) and/or standard error of mean (SEM).

### Western blot analysis, equipment and settings

Western blot (WB) analysis was done as before^59^. Cells were collected and lysed in RIPA Lysis and Extraction Buffer (Thermo Fisher Scientific), supplemented with 1% protease inhibitor Mix (Sigma Aldrich), 1% phenylmethanesulfonyl fluoride (PMSF) (Sigma Aldrich) and 10% PhosSTOP (Sigma Aldrich). The concentration of protein lysates was quantified using the Pierce™ BCA Protein Assay Kit (Thermo Fisher Scientific, Milan, Italy). Isolated proteins were separated by 4–15% precast Mini-PROTEAN® TGX Stain-Free™ Protein Gels (Bio-Rad, Segrate, Italy) and transferred to nitrocellulose membranes by Trans-Blot® Turbo™ Transfer System (Bio-Rad, Segrate, Italy). Membranes were incubated with the following primary antibodies diluted 1:1000 in blocking buffer (Bio-Rad, Segrate, Italy): rabbit anti-PARP-1, -BCL-XL, -caspase 3 (all Cell Signaling), -HOXA13 (Thermo, Milan, Italy) and -GAPDH (Santa Cruz, Milan, Italy) and mouse anti-β-catenin (Thermo, Milan, Italy)^59^. Secondary incubation was performed with goat anti-mouse/rabbit HRP-conjugate secondary antibodies (ImmunoReagents, Raleigh, USA). Protein bands detection was performed by Clarity Western ECL Substrate (Bio-Rad, Segrate, Italy) and membranes scanned using ChemiDoc™ MP Imaging system (Bio-Rad, Segrate, Italy). Proteins were visualized/quantified using a Chemiluminescence Plus kit (Thermo, Milan, Italy) and by Image Lab Software 4.0 (Bio-Rad, Segrate, Italy). Membranes were cut prior to hybridization with antibodies. Target protein acquisitions provided different settings of the Image Lab Software 4.0. In particular, the Chemi Hi Resolution application was firstly selected. Then, the mini-PROTEAN gel setting was selected for the imaging area. Exposure time was manually set and variated according to the target protein being considered. The acquisition of the molecular size marker was performed using the Stain Free Blot application and the mini-PROTEAN gel setting was selected for the imaging area. In this case, the time of exposure was manually set at 0.2 s. Acquisitions were modified using the Auto Scale setting of the Image Transform tool of ImageLab Software 4.0.

### Confocal immunofluorescence

Cells were grown on 13-mm coverslips, washed with PBS and fixed in methanol\acetone 1:1 for 7 min at − 20 °C. After PBS washing, cells were permeabilized with 0.1% Triton X-100 in PBS for 10 min at Room Temperature (RT). Cells were then incubated with mouse anti-β-catenin primary antibody (Thermo Fisher, Milan, Italy) in PBS containing 1% Bovine Serum Albumin (BSA) for 3 h at RT, washed with PBS and incubated with Alexa Fluor 488-conjugated secondary antibody (ThermoFisher, Milan, Italy) at a dilution of 1:400 in PBS containing 1% BSA for 1 h at 37 °C. Coverslips were mounted with mounting medium and DAPI reagent at RT, and images were acquired with a Olympus FV3000 confocal microscope equipped with a 60 × oil-immersion objective and processed by using the open-source Fiji software.

### Statistical analysis

Values were analyzed using the D'Agostino Pearson normality test, and parametric and nonparametric tests were applied according to normal and non-normal variables, respectively^60^. In particular, student *t* test or one/two-way analysis of variance (ANOVA test) were then used for normal distributions^61^, while Mann–Whitney *U* or Kruskal–Wallis tests were used for non-normal distributions [95% confidence interval (CI)]^62^. DdPCR experiments were performed on two biological replicates of epithelial cell lines and three biological replicates of cervical cancer cell lines as well as on all normal/CIN tissue specimens. Three technical replicates per each cell line/tissue sample were used for each ddPCR experiment. Transfections and related ddPCR experiments, colony formation and migration assays were performed on two biological replicates, i.e. HaCaT and NCTC cells, of which three technical replicates per experimental condition were used. Cell proliferation and immunofluorescence were evaluated in HaCaT cells with three technical replicates per experimental condition. Results are reported as mean value ± standard deviation of mean (SD) and/or standard error of mean (SEM). Spearman correlation coefficient r was used to evaluate correlations among miRNA/gene expressions. DdPCR data analyses were conducted using Quanta Soft software (Bio-Rad, Segrate, Italy). Spearman correlation coefficient r was used to evaluate correlations among miRNA/gene expressions. Statistical analyses were performed using Prism 8.0 statistical software (Graph Pad, La Jolla, USA)^63^. P < 0.05 were considered statistically significant.

### Ethics approval

Institutional Review Board approval was obtained from University Ferrara Hospital Ethical Committee (ID: 160986).

### Patient consent statement

Written informed consent was obtained from all subjects.

### Permission to reproduce material from other sources

We authorize the reproduction of material from other sources.

### Supplementary Information

Supplementary Figure S1.

Supplementary Information.

## Data Availability

All data generated or analyzed during this study are included in this article. Further enquiries can be directed to the corresponding author. Raw data will be shared upon request.

## Abbreviations

miRNA: MicroRNA
HOXA13: Homeobox A13
EMT: Epithelial-mesenchymal transition
