# Supplementary material for: Hsa-microRNA-1249-3p/Homeobox A13 axis modulates the expression of β-catenin gene in human epithelial cells
Source: Sci Rep. 2023 Dec 18;13:22872. doi: 10.1038/s41598-023-49837-0 (PMC10739948; doi:10.1038/s41598-023-49837-0)
Supplement: Supplementary file 1 — Supplementary Figure S1. [file 41598_2023_49837_MOESM1_ESM.docx]

**Supplementary files**

**
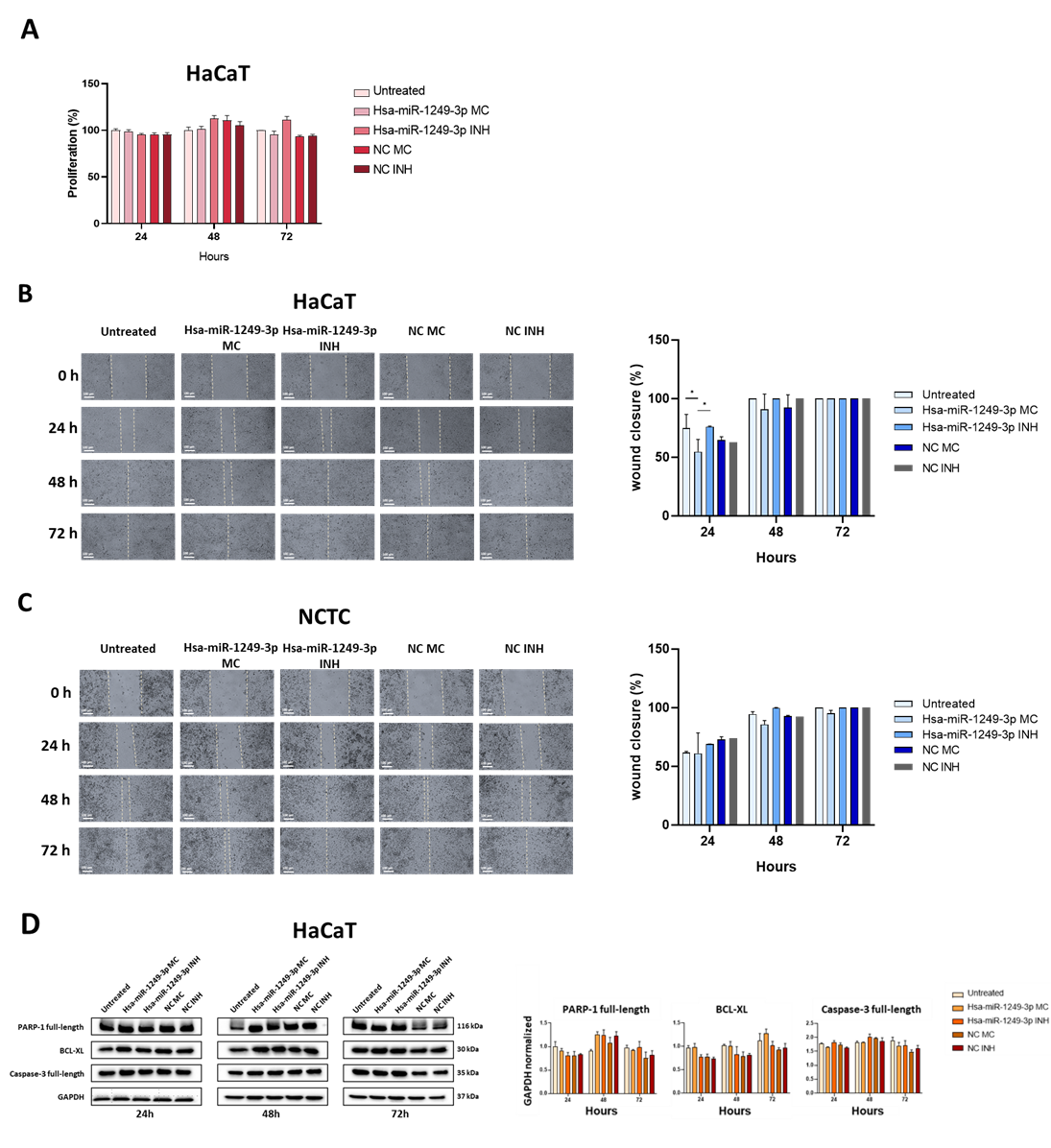
**

**Figure 1S. Cell proliferation, migration, and apoptosis evaluation in hsa-miR-1249-3p mimic, hsa-miR-1249-3p inhibitor, and negative controls of transfection HaCat and NCTC cells. A)** Cell proliferation was evaluated by WST-1 in HaCaT cells at 24, 48 and 72 h after transfection. Graphical data represent the mean % ± standard error of mean (SEM) of proliferation after transfection over untreated control values. **B, C)** Cell migration was evaluated by wound healing assays in HaCaT and NCTC cells at 24, 48 and 72 h after transfection. Graphical data represent the mean % ± standard error of mean (SEM) of wound closure after transfection over untreated control values. *p<0.05. **D)** Apoptosis was evaluated by western blot (WB) analysis at 24, 48 and 72 h after transfection in HaCaT cell line. WB analysis was performed by densitometry quantification of protein levels of PARP-1 full-length (116 kDa), Caspase-3 full length (35 kDa) and BCL-XL (30 kDa) and normalized to GAPDH (37 kDa). Results are shown as mean ± standard deviation (SD). All panels: MC: mimic, INH: inhibitor, NC: negative control.
