# Supplementary material for: Hsa-microRNA-1249-3p/Homeobox A13 axis modulates the expression of β-catenin gene in human epithelial cells
Source: Sci Rep. 2023 Dec 18;13:22872. doi: 10.1038/s41598-023-49837-0 (PMC10739948; doi:10.1038/s41598-023-49837-0)
Supplement: Supplementary file 2 — Supplementary Information. [file 41598_2023_49837_MOESM2_ESM.pdf]

HaCat Day 1

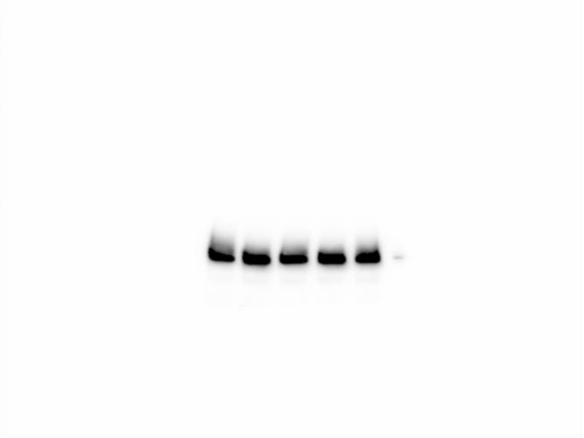

PARP-1 full length

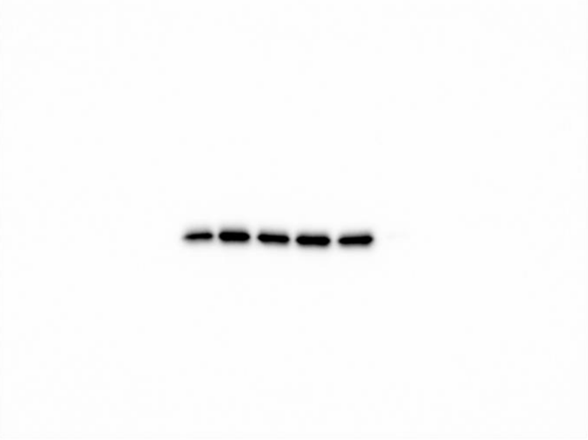

BCL-XL

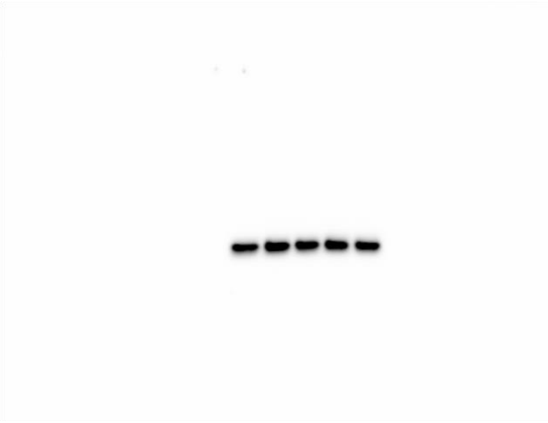

caspase-3 full length

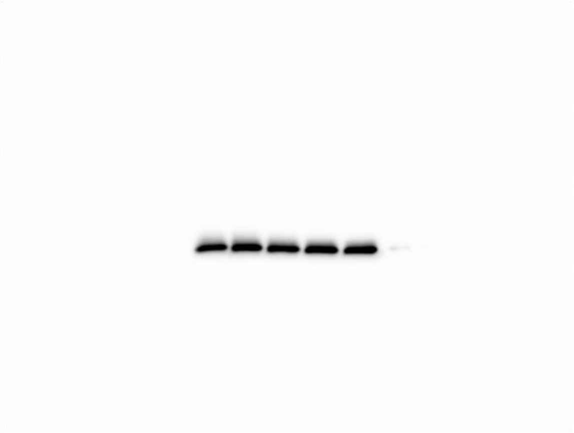

GAPDH 1

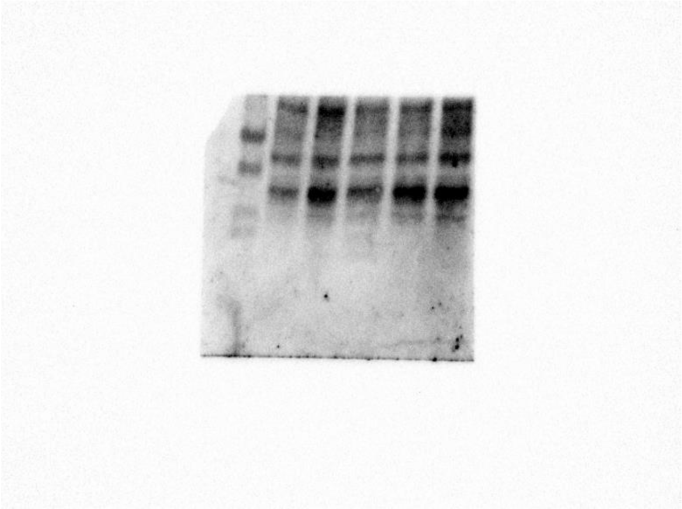

HOXA13

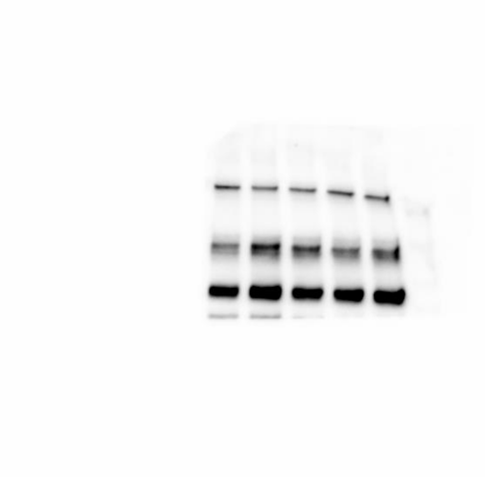

β-catenin

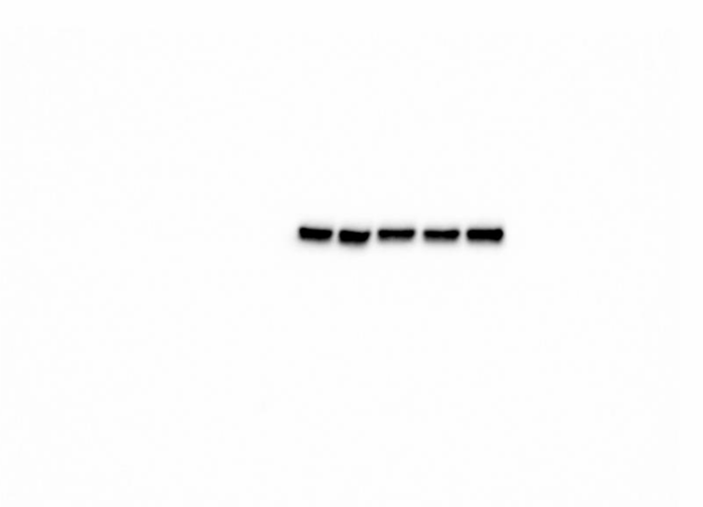

GAPDH 2

HaCat Day 1

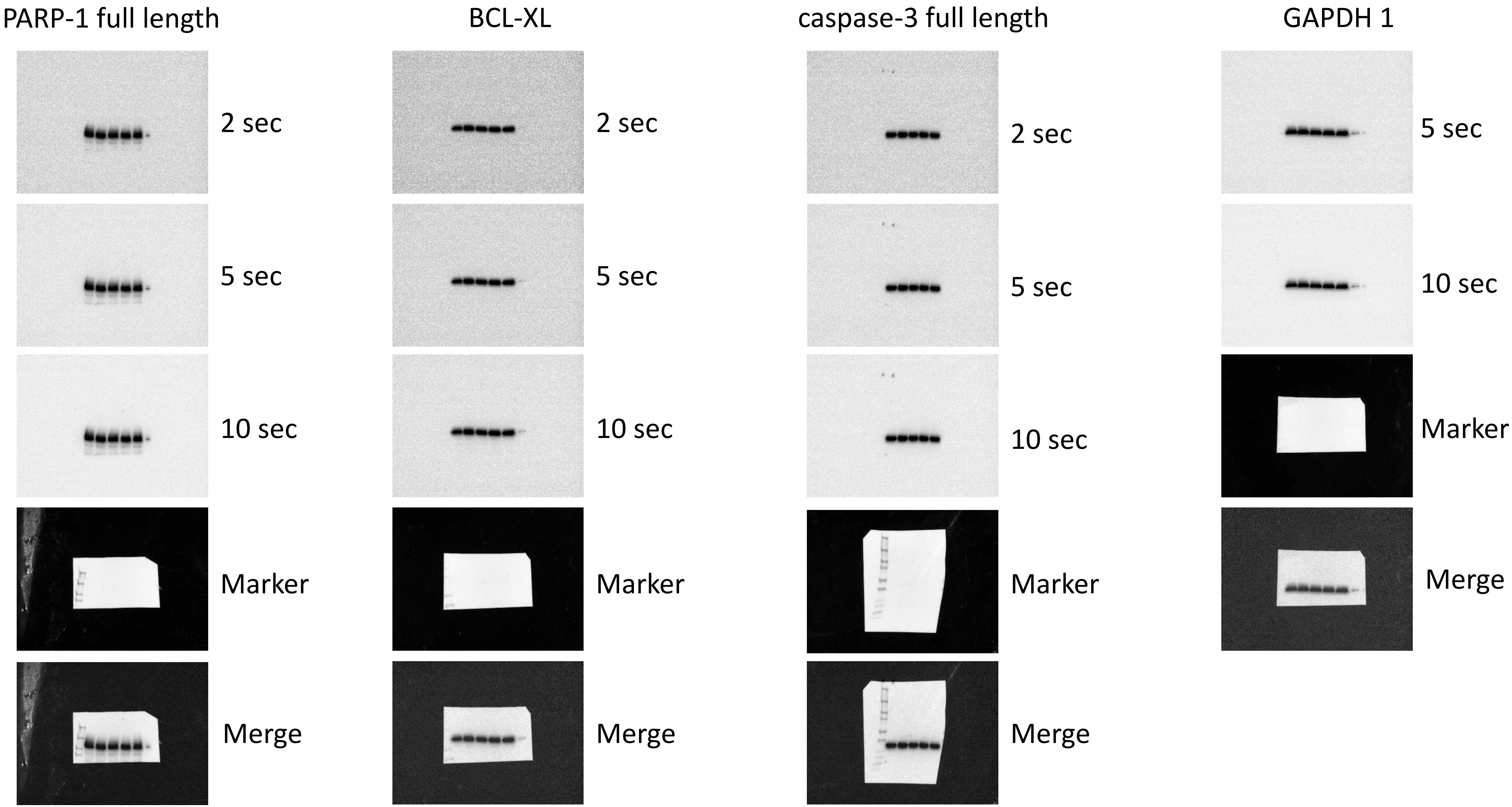

PARP-1 full length
BCL-XL
caspase-3 full length
GAPDH 1
2 sec
5 sec
10 sec
Marker
Merge
2 sec
5 sec
10 sec
Marker
Merge
2 sec
5 sec
10 sec
Marker
Merge
5 sec
10 sec
Marker
Merge

HaCat Day 1

HOXA13

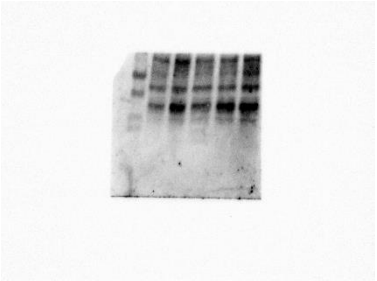

10 sec

β-catenin

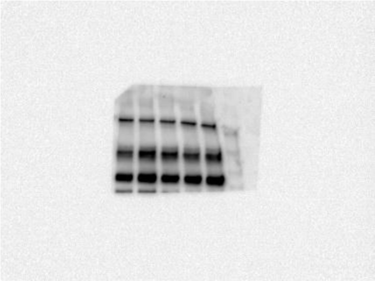

2 sec

GAPDH 2

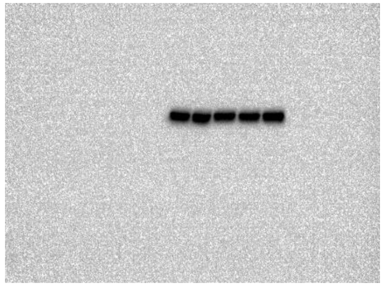

0.2 sec

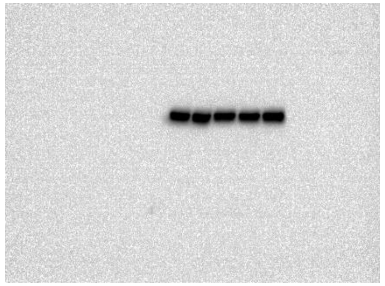

0.5 sec

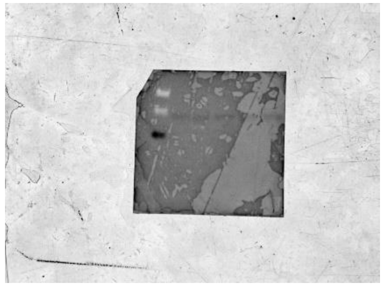

Marker

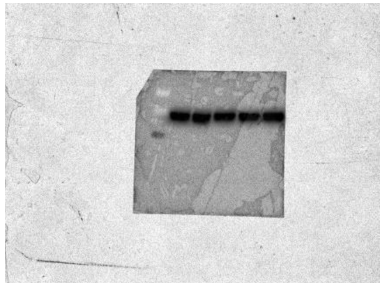

Merge

HaCat Day 2

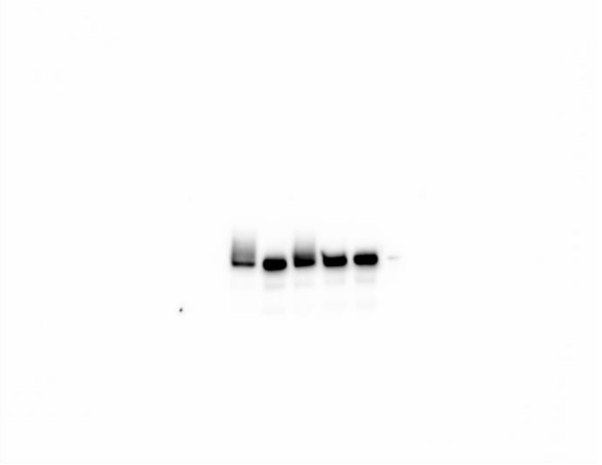

PARP-1 full length

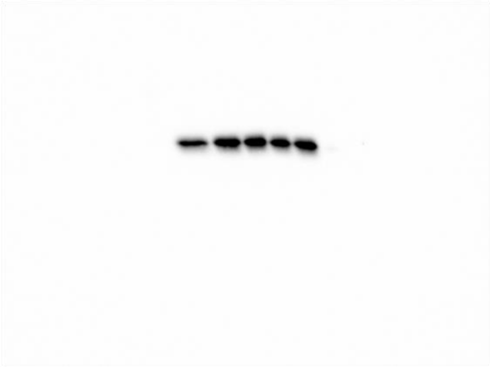

BCL-XL

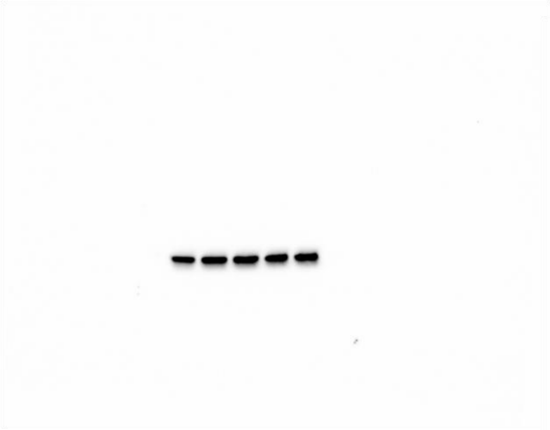

caspase-3 full length

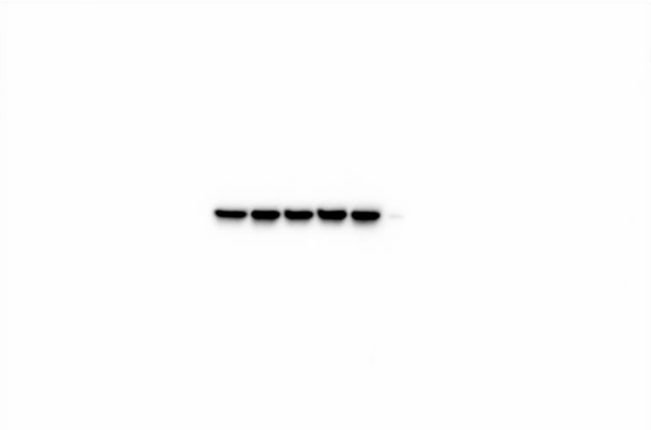

GAPDH 1

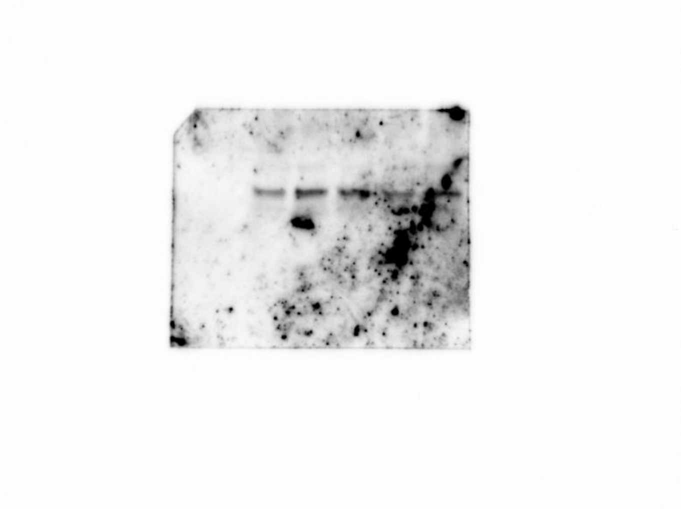

HOXA13

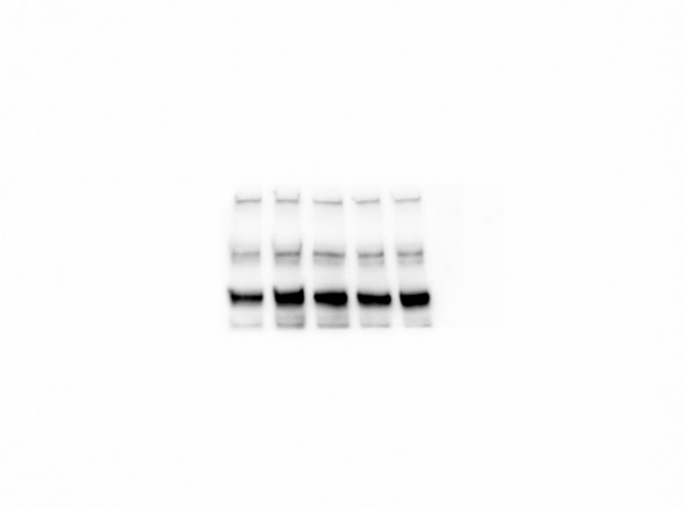

β-catenin

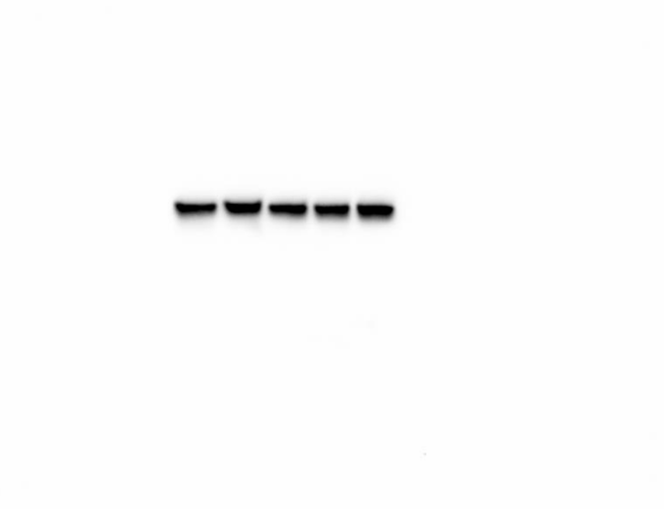

GAPDH 2

HaCat Day 2

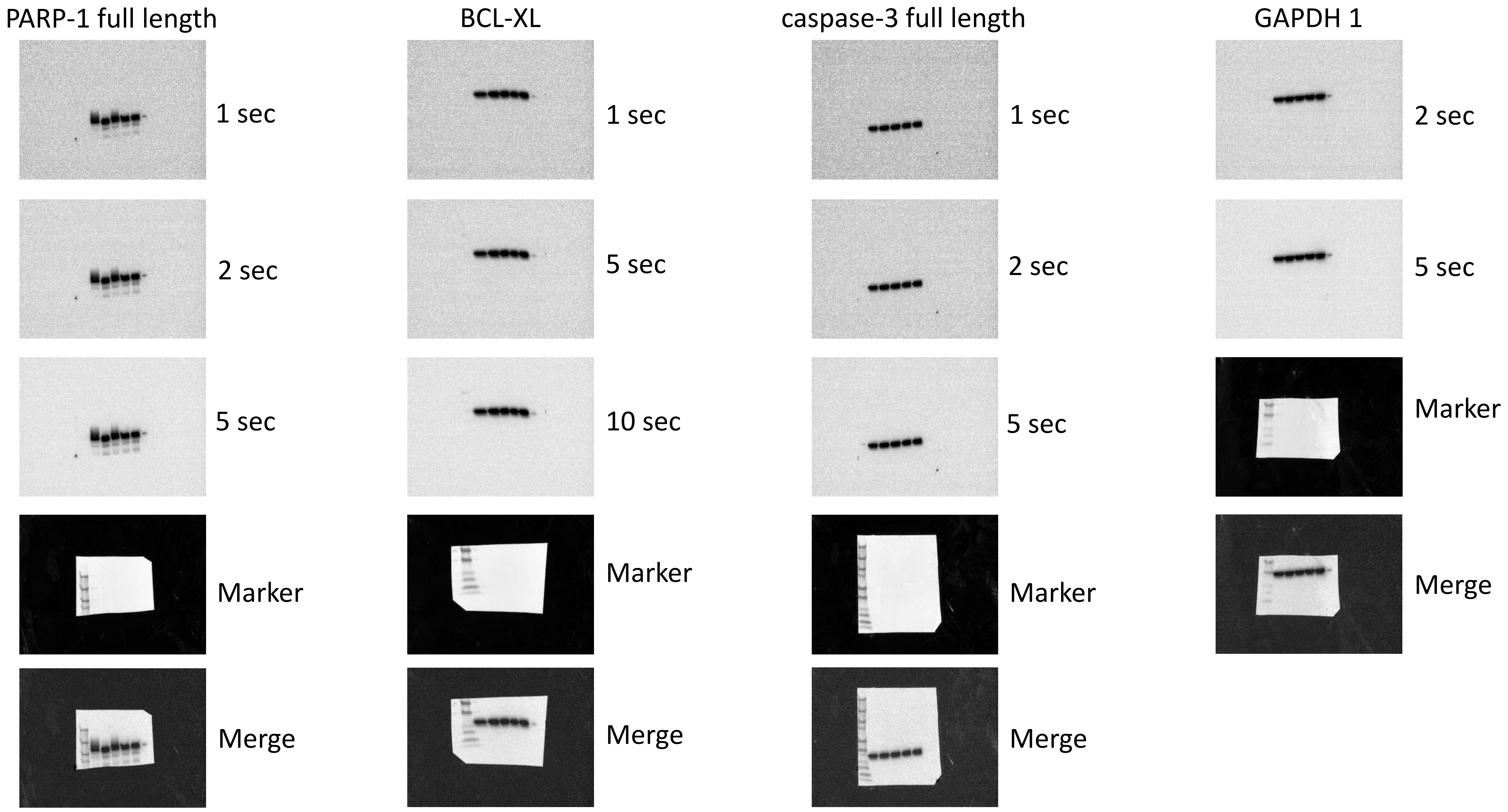
PARP-1 full length
BCL-XL
caspase-3 full length
GAPDH 1
1 sec
2 sec
5 sec
Marker
Merge
1 sec
5 sec
10 sec
Marker
Merge
1 sec
2 sec
5 sec
Marker
Merge
2 sec
5 sec
Marker
Merge

HaCat Day 2

HOXA13

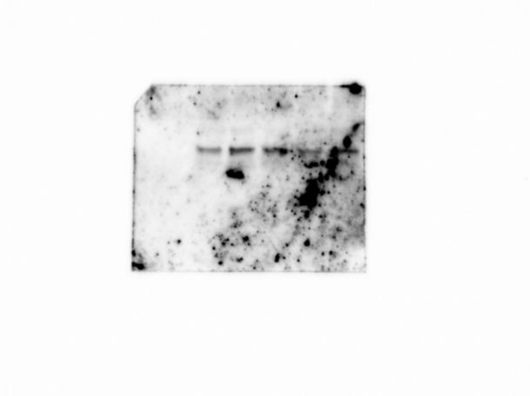

20 sec

β-catenin

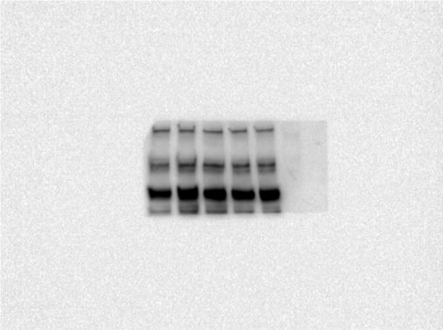

1 sec

GAPDH 2

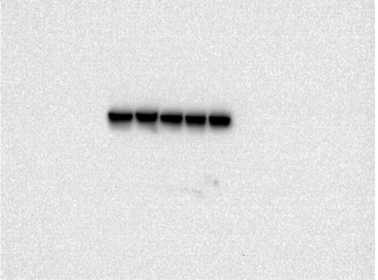

0.5 sec

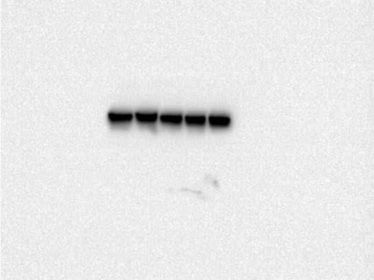

1 sec

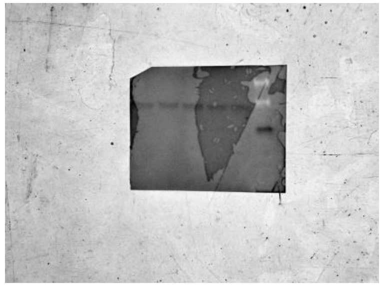

Marker

HaCat Day 3

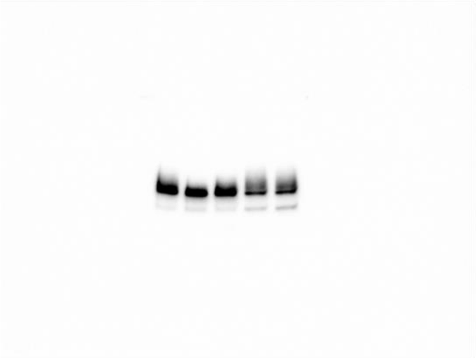

PARP-1 full length

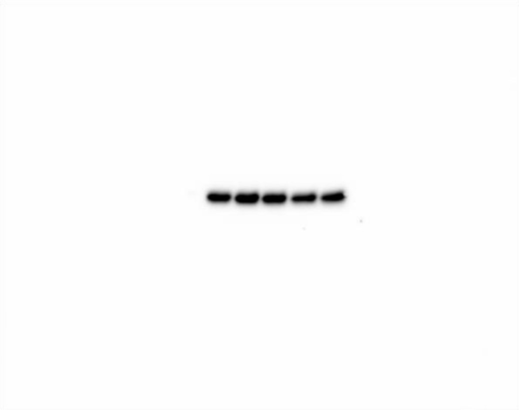

BCL-XL

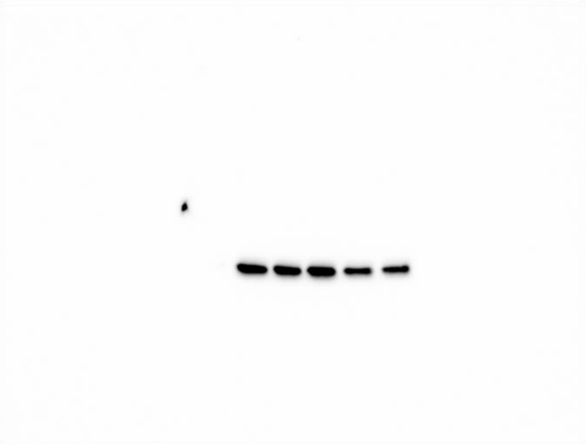

caspase-3 full length

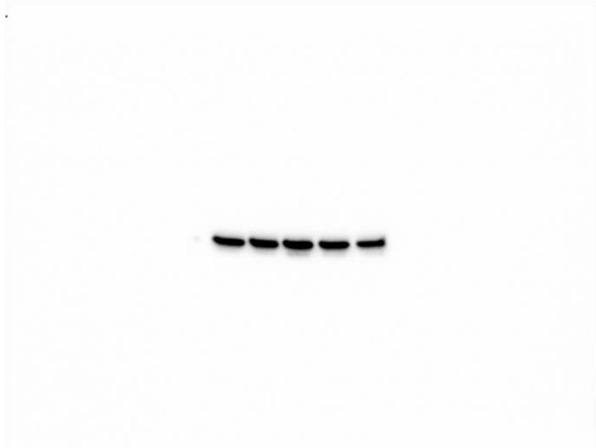

GAPDH 1

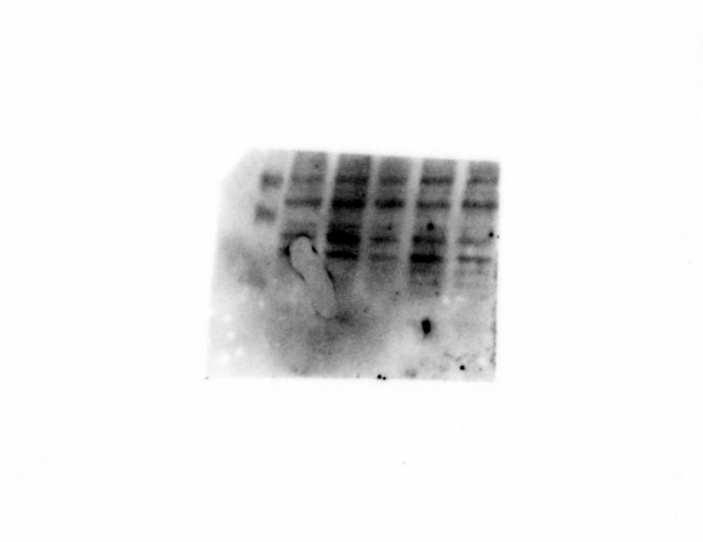

HOXA13

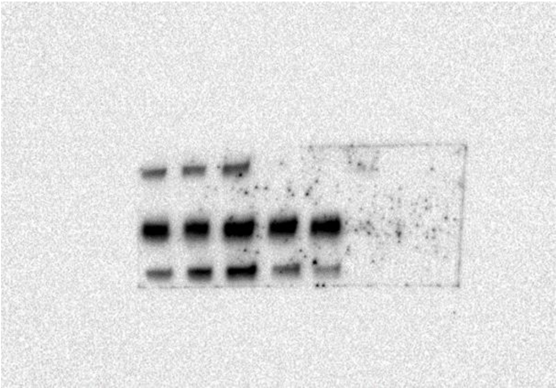

β-catenin

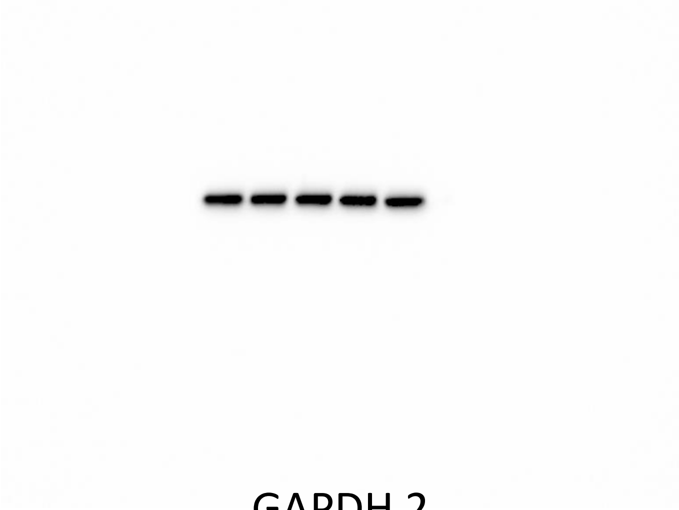

GAPDH 2

HaCat Day 3

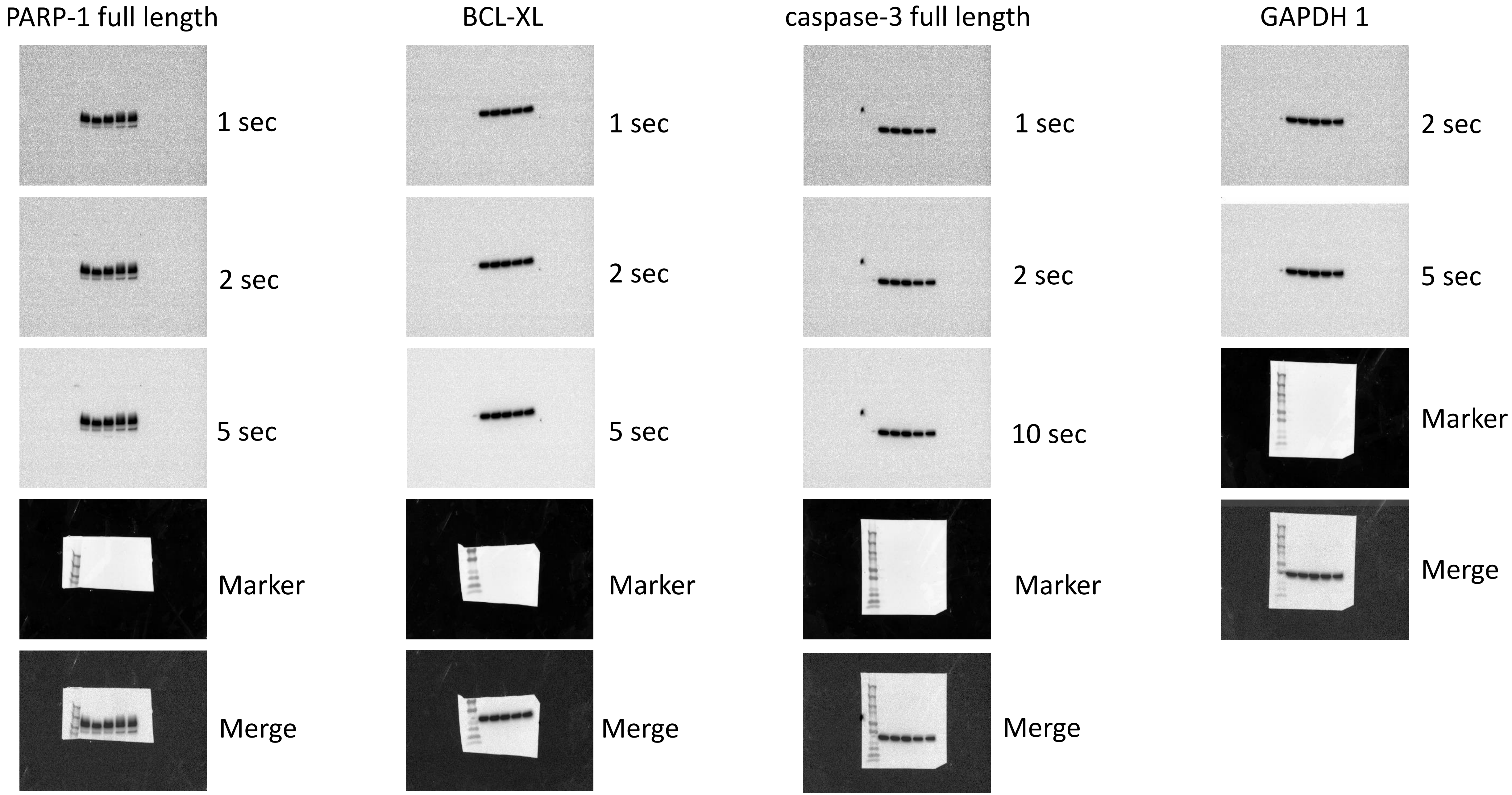
PARP-1 full length
BCL-XL
caspase-3 full length
GAPDH 1
1 sec
2 sec
5 sec
Marker
Merge
1 sec
2 sec
5 sec
Marker
Merge
1 sec
2 sec
10 sec
Marker
Merge
2 sec
5 sec
Marker
Merge

HaCat Day 3

HOXA13

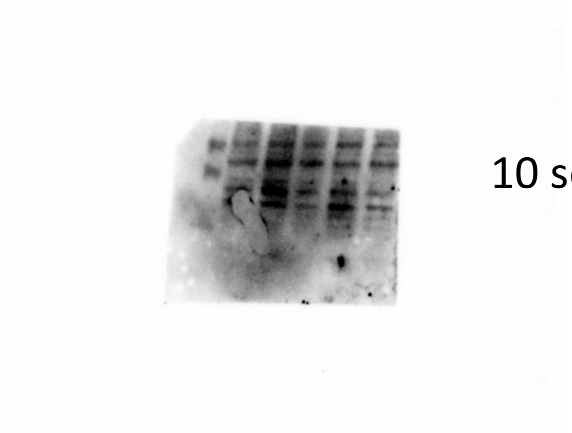

10 sec

β-catenin

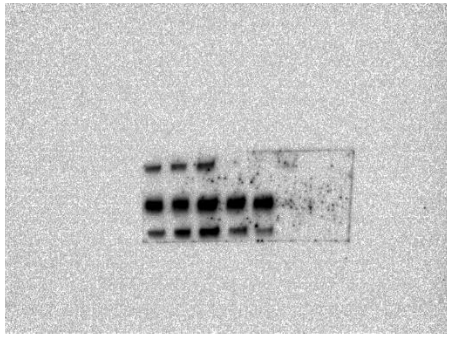

2 sec

GAPDH 2

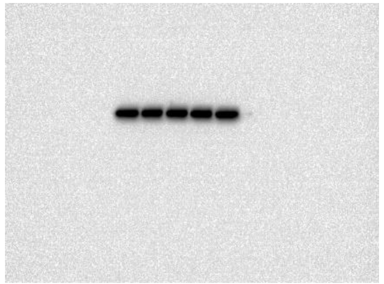

0.5 sec

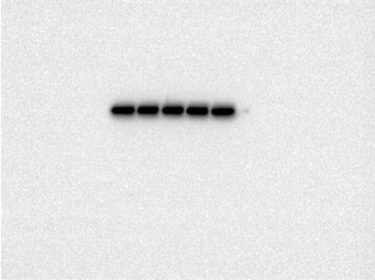

1 sec

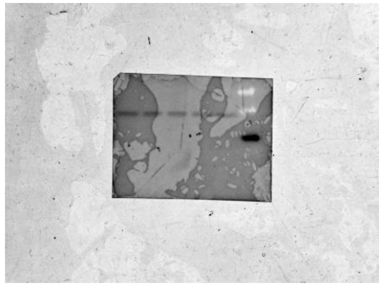

Marker

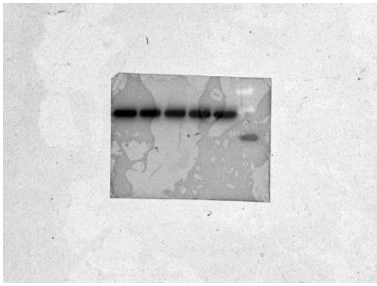

Merge

NCTC Day 1

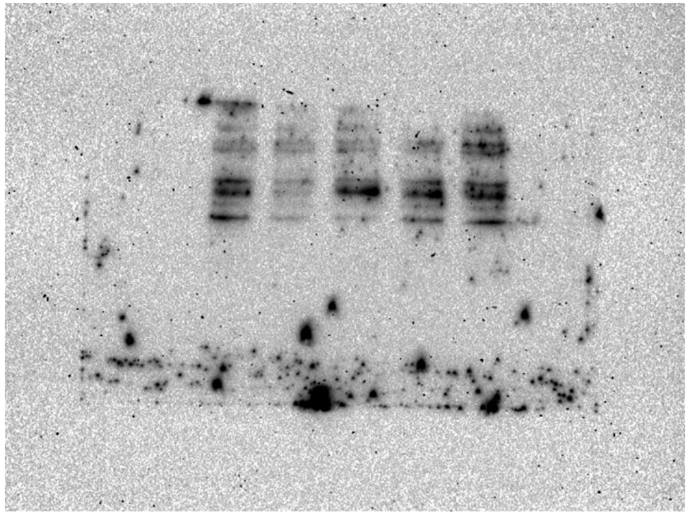

HOXA13

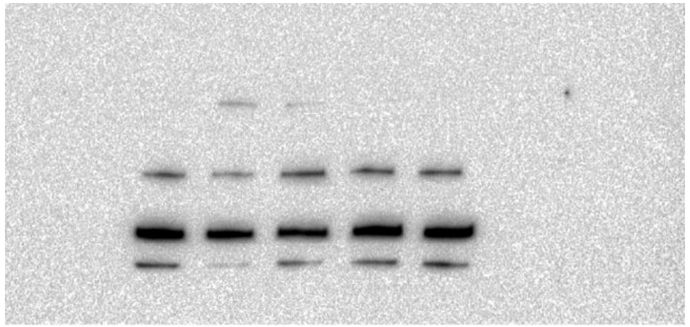

β-catenin

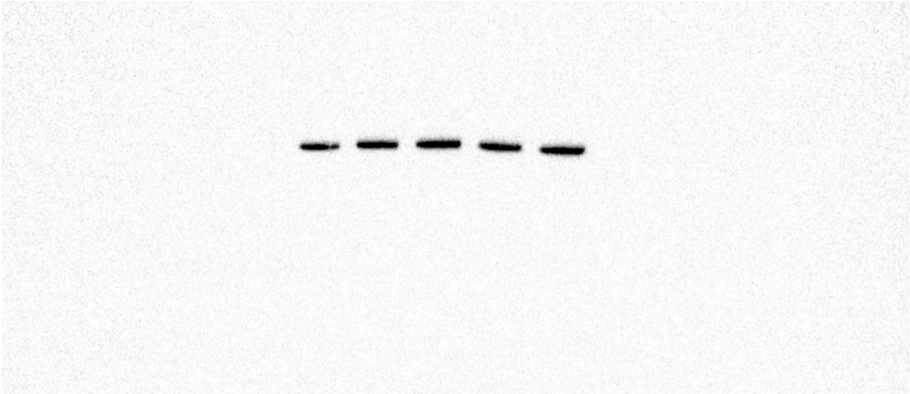

GAPDH

NCTC Day 1

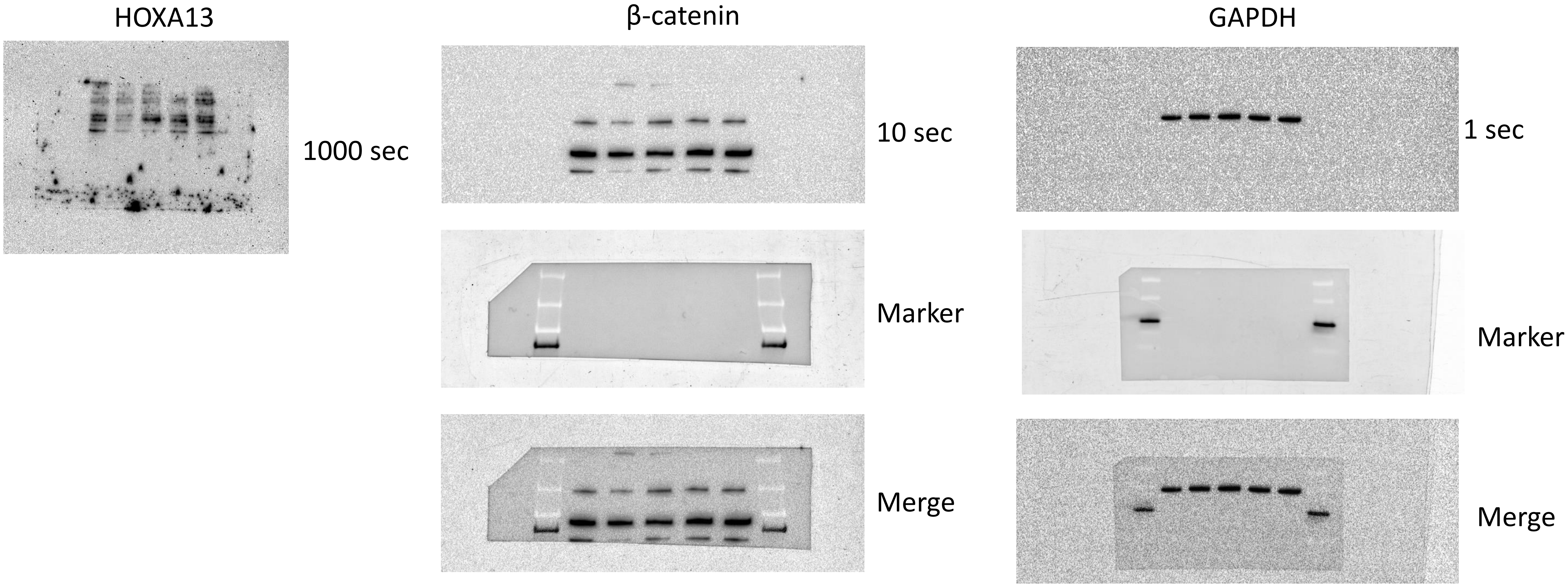
HOXA13
1000 sec
β-catenin
10 sec
Marker
Merge
GAPDH
1 sec
Marker
Merge

NCTC Day 1

GAPDH

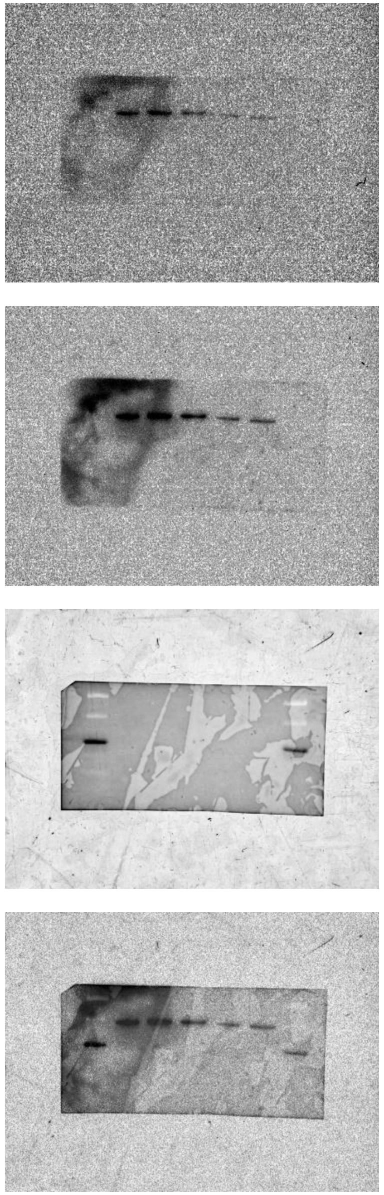

30 sec

120 sec

Marker

Merge

NCTC Day 2

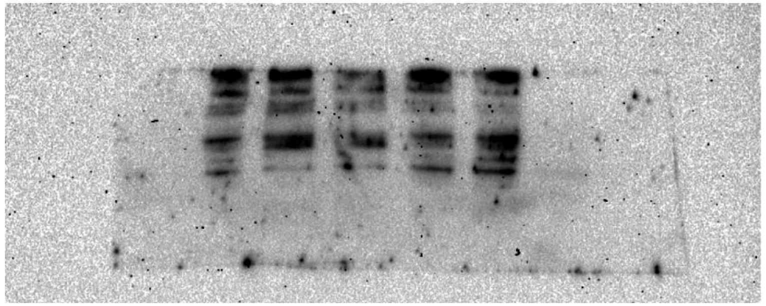

HOXA13

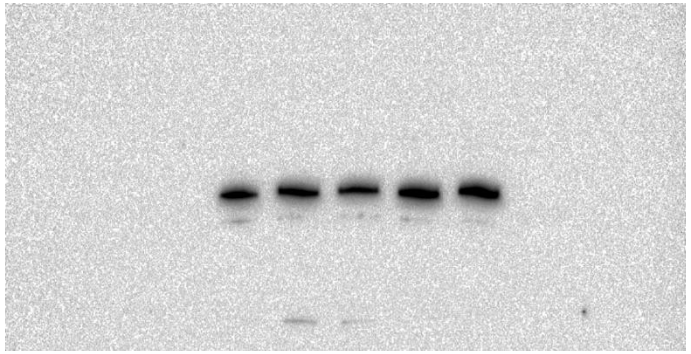

β-catenin

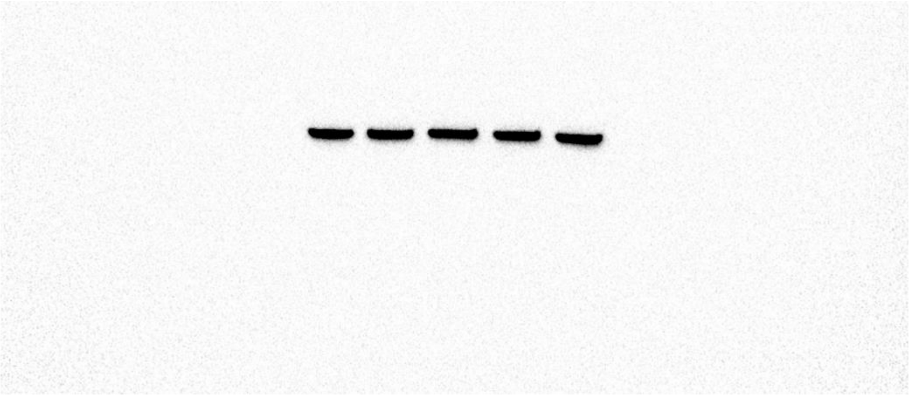

GAPDH

NCTC Day 2

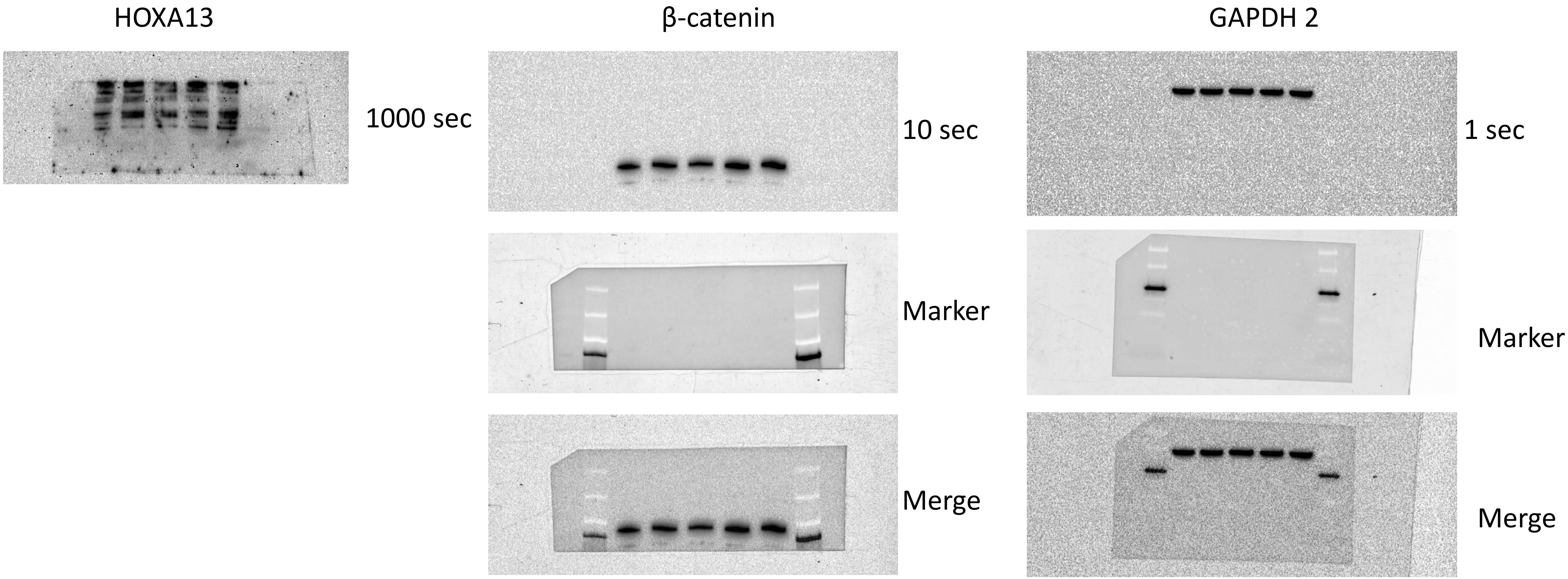
HOXA13
1000 sec
β-catenin
10 sec
Marker
Merge
GAPDH 2
1 sec
Marker
Merge

NCTC Day 2

β-catenin

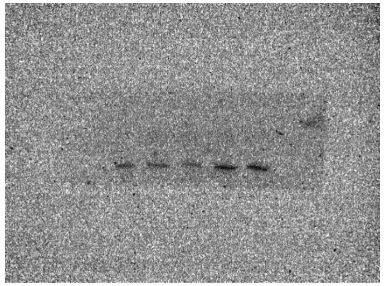

600 sec

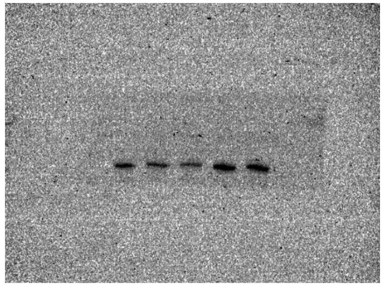

900 sec

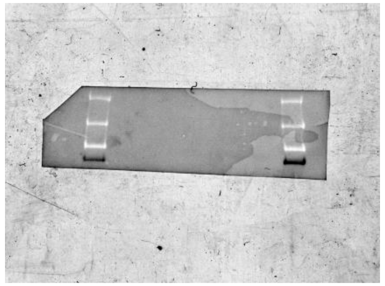

Marker

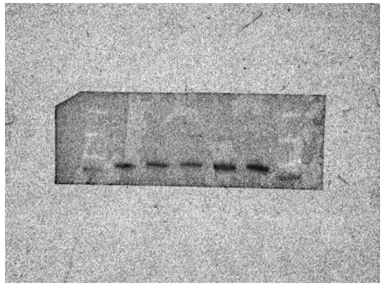

Merge

GAPDH

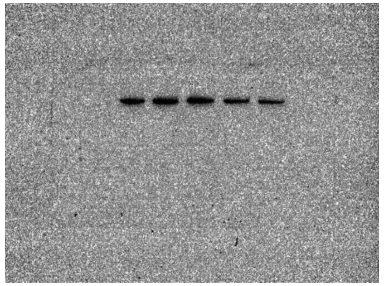

180 sec

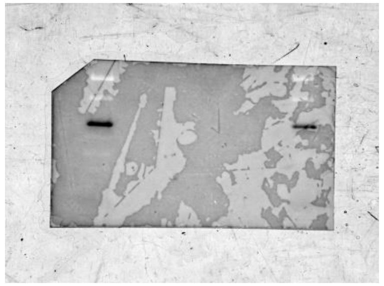

Marker

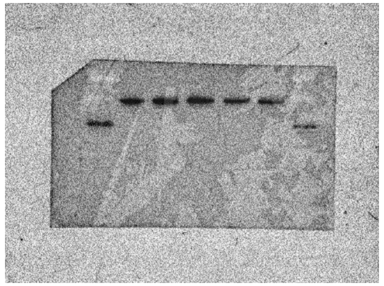

Merge

NCTC Day 3

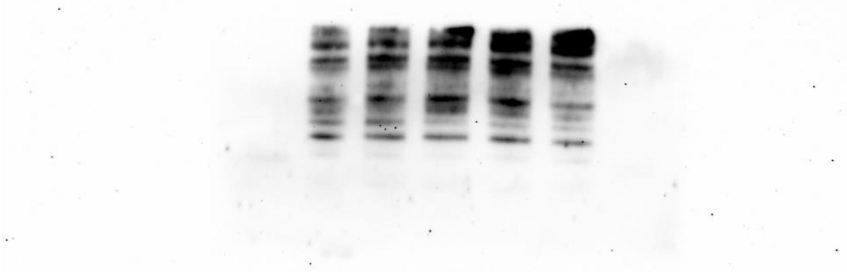

HOXA13

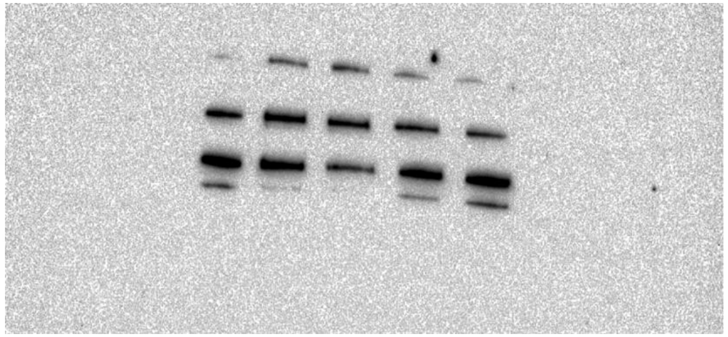

β-catenin

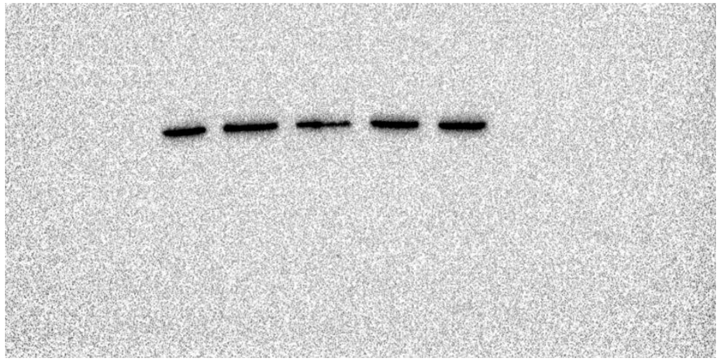

GAPDH

NCTC Day 3

HOXA13

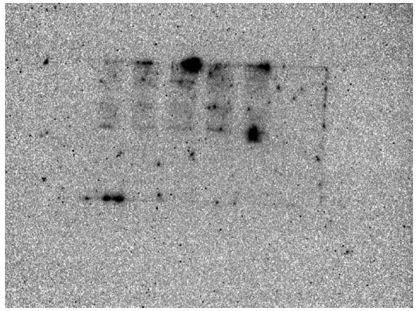

1000 sec

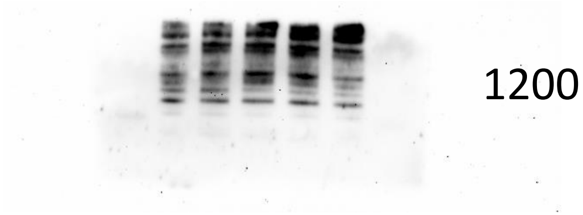

1200 sec

β-catenin

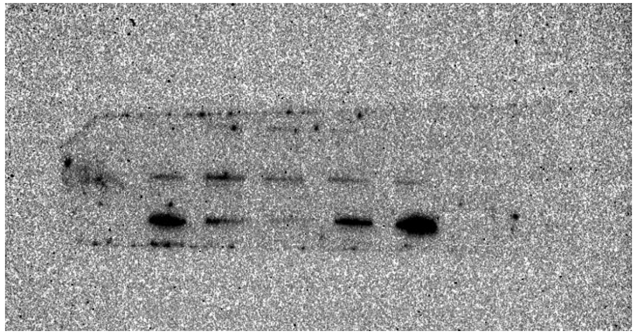

500 sec

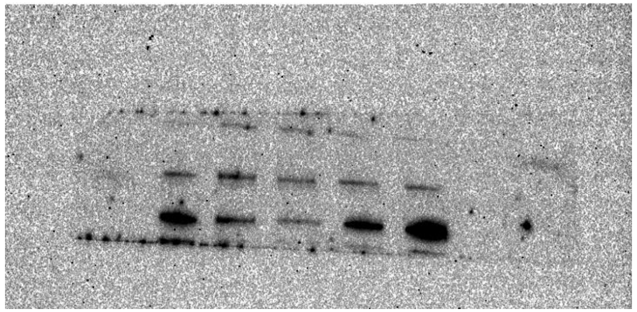

600 sec

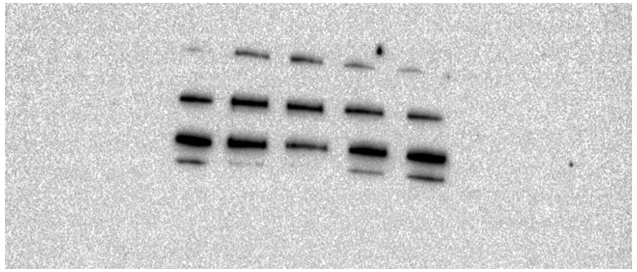

10 sec

GAPDH 2

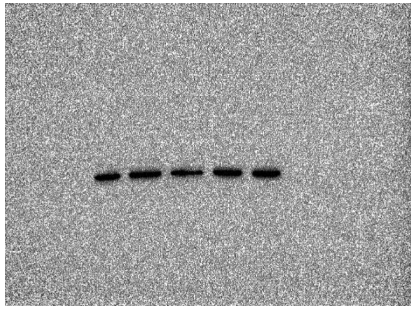

0.5 sec

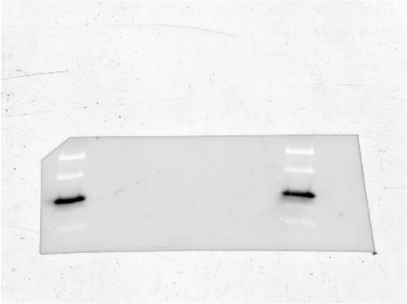

Marker

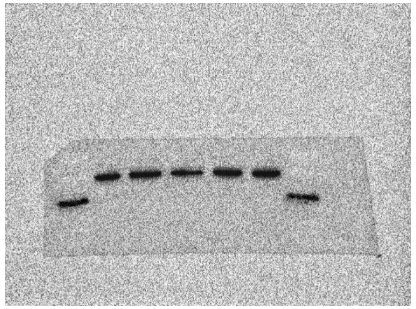

Merge
